# Shining light on clay–chromophore hybrids: layered templates for accelerated ring closure photo-oxidation†
†Electronic supplementary information (ESI) available. See DOI: 10.1039/c5sc02215k


**DOI:** 10.1039/c5sc02215k

**Published:** 2015-07-17

**Authors:** Ankit Jain, Amritroop Achari, Nivin Mothi, Muthuswamy Eswaramoorthy, Subi J. George

**Affiliations:** a Supramolecular Chemistry Laboratory , New Chemistry Unit , Jawaharlal Nehru Centre for Advanced Scientific Research , Jakkur P.O. , Bangalore 560064 , India . Email: george@jncasr.ac.in ; Email: eswar@jncasr.ac.in; b Nanomaterials and Catalysis Lab , Chemistry and Physics of Materials Unit , Jawaharlal Nehru Centre for Advanced Scientific Research , Jakkur P.O. , Bangalore 560064 , India

## Abstract

Templates with specific microenvironments have been long employed to facilitate specialized reactions. From enzymes to metal organic frameworks (MOFs), various systems have exerted their prowess to affect specific chemical reactions. Here we report, for the first time, the acceleration of a ring closure photo-oxidation reaction due to the specific structural constraints provided by layered materials. A stilbene derivative has been used as a prototype reactant and the di-hydrophenanthrene intermediate has been isolated and characterized *en route* to the complete photo-oxidation. Combining the gathered evidence, a possible mechanism for the chemical transformation has been proposed. Kinetic analysis showed that layered materials help to manipulate the rate of the electrocyclic ring closure and, in turn, accelerate the complete reaction sequence. The structural microenvironment induced by layered materials could be a unique platform to probe and stabilize a plethora of photo-oxidative reactions and intermediates.

## Abstract 2

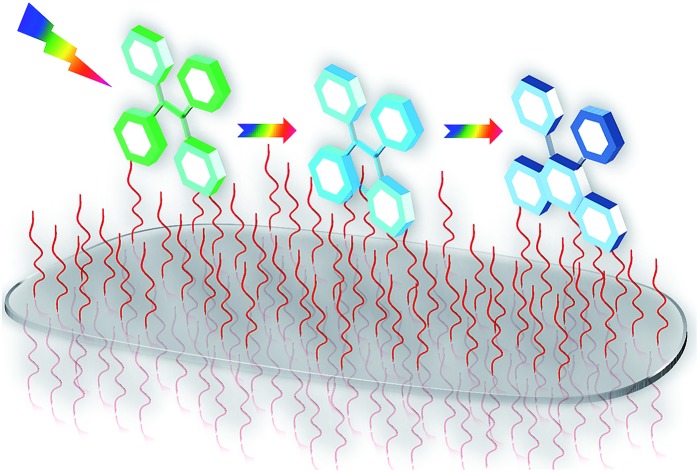
Clay–chromophore hybrids have been employed to critically affect the kinetic landscape of the ring closure photo-oxidation reaction.

## Introduction

Molecular templates have been long known to provide local environments for specific chemical reactions by virtue of their structural and functional prowess. Enzymes and DNA are some naturally occurring examples.^1^ The interior environment of polymers and artificial cage-like systems such as MOFs and their discrete versions have been explored for probing interesting reactions and intermediates.^2^,^3^ Unique chemical reactions have also been demonstrated by exploiting inorganic layered compounds with functional interlayer galleries,^4^ which can be considered as analogues of the aforementioned inorganic templates and thus can be explored for reactions susceptible to confinement effects.^5^ Layered compounds, such as clay, have been a subject of interest in a wide variety of fields, especially for studying the photo-physical properties of dye conjugates.^6^ Chemical reactions that are governed by light are the most susceptible to pre-organization. However, only a handful of light mediated chemical transformations have been carried out on clay surfaces and the ring closure photo-oxidation of stilbene systems has been one of the rarely investigated themes in clay microenvironments.^5^ Furthermore, the sensitizers employed for these photo-oxidation reactions (external sensitizers or inherent ions in the clay) photo-resulted in either [2 + 2] dimers or fission products of the ethylene bond.^7^ Ring closure photo-oxidation reactions in stilbene systems to form phenanthrene derivatives are synthetically important as they are one of the important steps in the synthesis of various poly-aromatic hydrocarbons.^8^ This reaction has also been studied in detail with a special impetus on trapping the di-hydrophenanthrene intermediate. The di-hydrophenanthrene derivative has been isolated only in anaerobic conditions and is a key step in controlling the rate of the overall reaction.^9^,^10^ In the reaction sequence, ring closure turns out to be the slower step and hence the rate determining step. As soon as di-hydrophenanthrene is formed it is oxidized to its phenanthrene analogue.^9^,^10^ Hence, increasing the rate of ring closure will enhance the rate of phenanthrene formation. Generally, these reactions are carried out in the presence of an oxidizing agent, such as 2,3-dichloro-5,6-dicyano-1,4-benzoquinone (DDQ) or I_2_, owing to the slow photo-chemical reaction rate.^11^ It has therefore been a challenge to look for a strategy to enhance the rate of such reactions photo-chemically.

In this manuscript we, for the first time, observe that the kinetic pathways of organic photo-reactions can be modulated on layered inorganic templates. Photo-reactions on stilbene derivatives have been a research topic for decades, however, it is shown for the first time in this manuscript that its ring closure photo-oxidation kinetics can be biased while it is conjugated on an inorganic clay surface. To add to the novelty of the manuscript, during the photo-reaction process, we have isolated a di-hydrophenanthrene derivative as an intermediate, which has proved to be a challenging task in the past. These derivatives are generally very unstable and their complete characterization, as done in this manuscript, is very rare.

## Results and discussion

### Design strategy

**Fig. 1:**
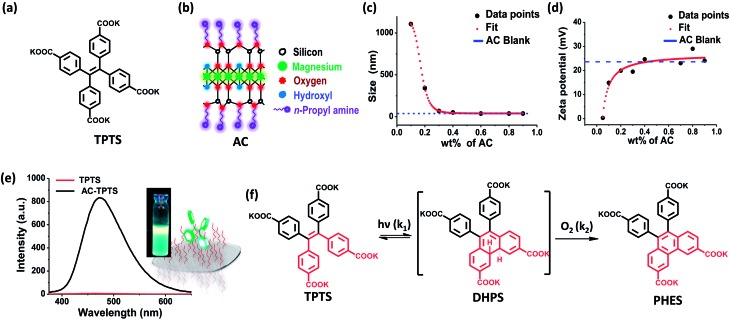
(a) Molecular structure of **TPTS**, (b) schematic showing the structure of amino clay (**AC**), (c) and (d) DLS and zeta potential trends of **TPTS** (10^–4^ M) with various weight percentages of **AC**. (e) Emission spectra showing the enhanced emission from **TPTS** (10^–4^ M) on binding to **AC** (0.9 wt%), this dye conjugated state has been designated as **AC-TPTS** (*l* = 1 cm, *λ*_exc_ = 350 nm, water) (inset shows a photograph of a 365 nm illuminated solution of the clay–dye conjugate alongside the respective schematic). (f) Reaction sequence showing the expected photo-oxidation reaction of **TPTS** (the stilbene moieties are marked in red). *k*_1_ and *k*_2_, mentioned above the arrowheads, are the forward rate constants.

The inorganic template we have used to construct the microenvironment for the chemical modification is amino clay (**AC**).12*a* It has a structure analogous to 2 : 1 trioctahedral smectite with an approximate composition of R_8_Si_8_Mg_6_O_16_(OH)_4_, where R stands for covalently linked aminopropyl chains (Fig. 1b). These clays do not have sensitizing action because of the absence of metal ions, such as Al^3+^ and Fe^3+^.^12^

Moreover, the optically transparent nature of **AC** facilitates photochemical reactions and its high dispersity helps maintain the reaction mixture as homogeneous. **AC** when mixed in water undergoes exfoliation, resulting in a clear solution, owing to the spontaneous protonation of the amine groups on the surface. Recently, many possibilities that can arise from the use of amino clay (**AC**) as a soft hybrid for various photo-physical prospects have been explored.^12^

Tetraphenylethylene (**TPE**) and its derivatives are stilbene analogues, and have a rich history of photo-physical and photo-chemical phenomena.^14^ With regards to the photo-physical properties, they are known to show a higher quantum yield of emission once their rotating phenyl propellers are stopped from moving.^15^ Enhanced emission due to restricted rotation has been used extensively for applications in opto-electronics and fluorescent probes.^15^ On the other hand, TPEs, being derivatives of stilbenes, also undergo unique photo-chemical modifications, such as photo-oxidation, which yields the phenanthrene derivative after a [4 + 2] electrocyclic ring closure.^9^ We envisaged that conjugating TPE moieties on clay could result in restricted rotation of the phenyl rings, which would enhance the probability of ring closure. Considering the nature of our inorganic template (positively charged amino clay), a negatively charged TPE derivative was selected, *i.e.* tetraphenylethylene tetra potassium salt (**TPTS**) (Fig. 1a). **TPTS** was synthesized according to the known procedure^16^ and the molecule was characterized by NMR spectroscopy and mass spectrometry (MS) (Fig. S1–S3†). **TPTS** has been studied in the past for its restricted rotation in MOFs by Dincă *et al.*^17^ The photochemistry of this molecule, however, has not been explored. Considering the classic model of electrocyclic ring closure, photo-oxidation of **TPTS** is expected to proceed to form the phenanthrene derivative (**PHES**) *via* the 4,4′-di-hydrophenanthrene intermediate (**DHPS**) (Fig. 1f).^9^

### Clay–dye conjugation

Preliminary experiments suggested that **TPTS** when conjugated with **AC** results in restriction of the rotation of the phenyl rings. The flattening of the ^1^H-NMR peaks and the emission enhancement in the clay–dye conjugate (**AC-TPTS**) (Fig. 1e) strongly support the restricted rotation of the phenyl rings on clay surfaces (Fig. S4†). To further understand the characteristics of the clay–dye conjugate, various wt% values of **AC** were added to a fixed concentration of aq. **TPTS** (10^–4^ M) and their dynamic light scattering (DLS) and zeta potential data were recorded (Fig. 1c and d). It can be clearly seen that beyond the neutralization point in the zeta potential (0 mV at 0.03 wt%), the size and charge density of the conjugate approach those of single **AC** sheets. Therefore, to study the photochemistry of **TPTS** on a clay surface, the concentration of **AC** was fixed at 0.9 wt% as this ensures complete binding and a highly homogenous dispersion.

### Photochemical studies

**Fig. 2:**
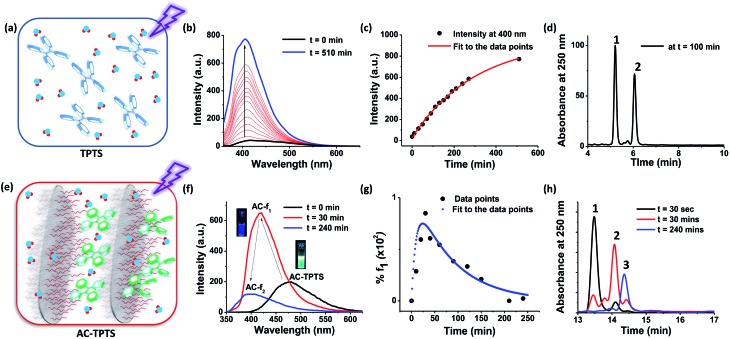
(a)–(d) The top panels represent the photo-oxidation of nascent **TPTS**, while (e)–(h) (the bottom panels) represent the photo-oxidation of the **TPTS** and **AC** conjugate in aqueous solution ([**TPTS**] = 10^–4^ M, *l* = 1 cm, *λ*_exc_ = 350 nm, water). (a) Schematic representation of the irradiation of nascent **TPTS** molecules in water, (b) time dependent emission spectra of the photo-oxidation of an aqueous **TPTS** solution under 254 nm irradiation, (c) fluorescence intensity trace at 400 nm of the photo-oxidation profile of **TPTS**, (d) HPLC chromatograph after 100 min of irradiation probed at 250 nm absorption,^13^ (e) schematic representation of the irradiation of the **TPTS** and **AC** conjugate, (f) time dependent emission spectra of the photo-oxidation of the **TPTS** (10^–4^ M) and **AC** (0.9 wt%) conjugate under 254 nm irradiation (inset shows photographs of 365 nm illuminated solutions of **AC-TPTS** and **AC-f_1_**), (g) fluorescence intensity trace at 400 nm of the photo-oxidation profile of the **TPTS** and **AC** conjugate, and (h) HPLC chromatographs after various times of irradiation probed at 250 nm absorption.^13^

Before analysing the photo-chemical characteristics of **AC-TPTS**, we sought to understand the chemistry of an aqueous **TPTS** solution under light (top panel, Fig. 2). A 10^–4^ M solution of **TPTS** was irradiated under a 254 nm lamp (Fig. 2a and b). Initially at *t* = 0, the emission is quenched due to non-radiative decay processes.^8^ With time, the evolution of an emission with *λ*_max_ at 404 nm could be observed (Fig. 2b), signifying the formation of the phenanthrene derivative. The intensity of the band at 400 nm was plotted against time, which showed a monotonic increase in intensity (Fig. 2c). The reaction took around 750 min to complete. Liquid chromatography-mass spectrometry (LC-MS) analysis of a 100 min aliquot showed two peaks (marked as 1 and 2 in Fig. 2d). The UV/vis absorption spectra and MS of fraction 1 corroborated with the starting material (**TPTS**), while fraction 2 pertained to **PHES**, as evident from the [M – 2] ion in the MS, signifying the loss of two protons due to oxidative ring closure (Fig. S5 and S6†). Time dependent ^1^H-NMR measurements were made of the irradiated sample and visual interpretation of the ^1^H-NMR spectra showed signs of a *C*_2_-symmetric species being formed, which also confirms the existence of the phenanthrene derivative (Fig. S7†). Knowing that the chemistry involved in the photo-conversion of stilbene derivatives to phenanthrene is a two-step process (Fig. 1f), the observation of a monotonic conversion into the final product points to the case of a series of reactions with *k*_2_ > *k*_1_. Considering the fact that the di-hydrophenanthrene intermediate is highly susceptible to oxidation, the hypothesis seems reasonable.^9^ Therefore, the evolution of phenanthrene can be fit into a specific case for reactions in series and the rate constant can be extracted (Fig. S8†). However, from the steady state data, only the slower reaction step (*k*_1_), *i.e.* the rate constant involved in the electrocyclic ring closure, can be extracted (Fig. S9†). After fitting to an appropriate function, *k*_1_ was found to be 3.7 × 10^–3^ s^–1^.

**Fig. 3:**
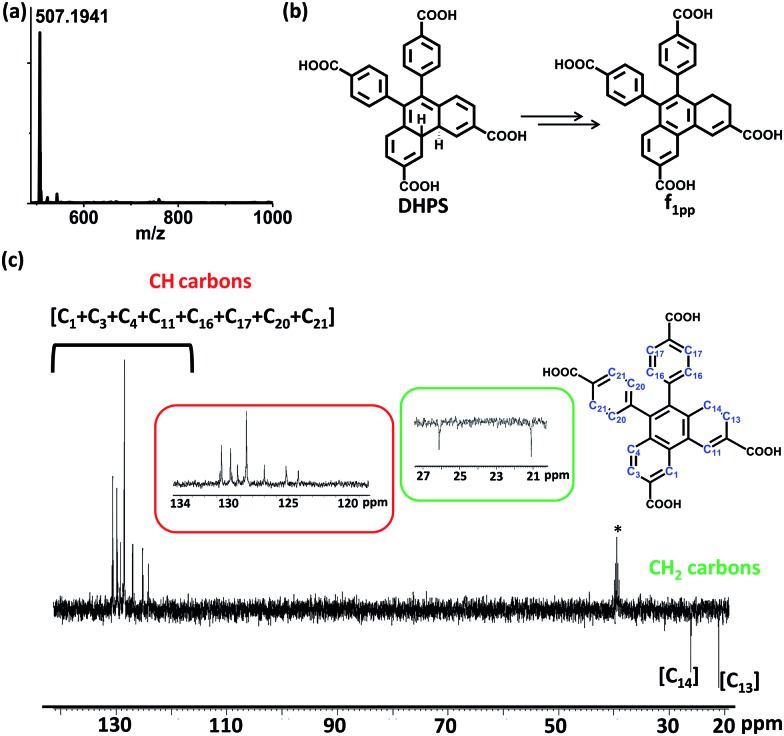
(a) The ESI-HRMS of **f_1pp_** in negative ion mode, (b) chemical transformation of **DHPS** to **f_1pp_**, and (c) the DEPT spectrum of **f_1pp_** in CD_3_OD (insets show zoomed in regions (120–134 and 21–27 ppm) and the **f_1pp_** structure with designated –CH and –CH_2_ carbons, *signifies the DMSO-d_6_ peak).

Interestingly, on the other hand, the clay–dye conjugate (**AC-TPTS**) (Fig. 2e) when irradiated with 254 nm light showed the progression to the final photoproduct *via* a two-step process, as evident from the corresponding fluorescence changes (Fig. 2f and g and S10†). The initial state **AC-TPTS** (*λ*_max_ = 475 nm) after 30 min went to a state **AC-f_1_** (*λ*_max_ = 420 nm) and then finally after 240 min to **AC-f_2_** (*λ*_max_ = 400 nm). The **AC-TPTS** to **AC-f_1_** transition was rapid and reached a maximum at around 30 min. The transformation of **AC-f_1_** to **AC-f_2_** was rather slow and took another 210 min to complete (Fig. 2f). Remarkably, the photo-oxidation was complete in 240 min, which is one third of the time taken without **AC**. The compositions **f_1_** and **f_2_** were extracted from the clay conjugates (**AC-f_1_** and **AC-f_2_**, respectively) and were analysed by LC-MS (Fig. 2h and S11–S14†). The LC-MS of **f_2_** showed the formation of phenanthrene (**PHES**, marked with 3 in Fig. 2h). UV/vis absorption spectroscopy and electrospray ionization-mass spectrometry (ESI-MS) performed on this fraction also corroborated with the conclusion that **f_2_** is predominantly a phenanthrene derivative (**PHES**) (Fig. S14†). The LC-MS of the **AC-TPTS** extract after 30 seconds of irradiation mainly comprised the starting material (**TPTS**, marked as 1 in Fig. 2h and S14†). It did, however, show traces of an intermediate peak (retention time (r.t.) = 14 min), which eventually became the major peak in fraction **f_1_** after 30 min (marked with 2 in Fig. 2h). The chromatogram after 30 min also contained a minor peak (r.t. = 13.8 min), which, due to the low yield, could not be further analysed. Interestingly, the UV/vis absorption spectrum of this fraction showed a red shifted band (366, 377 nm) as compared to the starting material (330 nm) (Fig. S13†). ESI-MS analysis, however, showed that the mass of this intermediate was the same as that of the starting material (Fig. 3a and S14†). This evidence hints towards **f_1_** being composed of a di-hydrophenanthrene derivative. Attenuated total reflectance-infra red (ATR-IR) analysis carried out on the intermediate showed a shift of the C–H stretch to a lower wavenumber (2962 to 2924 cm^–1^) as compared to the **TPTS** acid, which also signifies the introduction of a sp^3^ hybridized C–H bond (Fig. S15†).

Since di-hydrophenanthrene derivatives have rarely been isolated and characterized, we decided to do extensive characterization of these species.^10^ To our surprise, the isolated derivative from fraction **f_1_** was mainly the 1,2-di-hydro derivative (**f_1pp_**) instead of the 4,4′-di-hydro derivative (**DHPS**, Fig. 3b) (*vide infra*). Owing to the intermediate’s good oxygen tolerance, it was possible for us to carry out extensive NMR characterization and elucidate its structure. Distortionless enhancement by polarization transfer (DEPT) analysis showed that **f_1pp_** comprises –CH_2_ carbons, as shown by the negative peaks in Fig. 3c (peaks in the 20–30 ppm region). Counting the number of –CH and –CH_2_ peaks, it became evident that the di-hydro derivative is indeed an isomerized counterpart of **DHPS**.

**Fig. 4:**
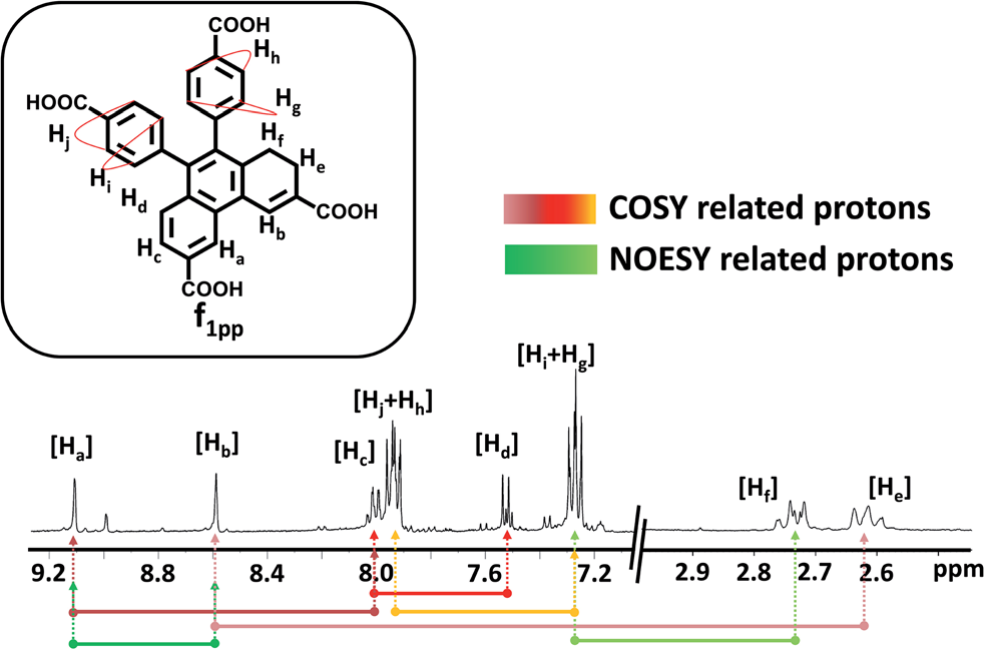
^1^H-NMR spectrum of **f_1pp_** in CD_3_OD (inset shows the molecular structure of **f_1pp_** with designated protons). The lines and arrows below the ^1^H-NMR spectrum show a summary of the ^1^H correlations observed in **f_1pp_**.

Visual interpretation of the ^1^H and ^13^C-NMR spectra showed that the spectra deviate from the *C*_2_-symmetric spectra obtained for phenanthrene (Fig. 4 and S16–S22†). Moreover in the ^1^H-NMR spectrum, two triplets could be clearly seen in the low field region of 2.6–2.8 ppm. From the heteronuclear correlation (HETCOR) spectrum, these ^1^H-peaks around 2.6–2.8 ppm could be correlated with the –CH_2_ peaks as determined by DEPT (Fig. S23†). The remainder of the –CH correlations were also made once the 2D spectrum was analysed (Fig. S24†). Having understood the carbon skeleton of the molecule, we went ahead to decipher the proton correlation existing in **f_1pp_**.

^1^H-correlation spectroscopy (COSY) helped in understanding the through bond proton connections of the molecule (**f_1pp_**, Fig. 4 and S25–S27†). Protons H_i_ and H_g_ correlated with H_j_ and H_h_, respectively, confirming the nascent aromatic structure of the part of the molecule that did not take part in the photo-reaction (Fig. 4 and S25†). Proton H_c_ correlated with H_d_, confirming the presence of an aromatic system in the modified part of the molecule (Fig. 4 and S25†). The proton designated as H_a_ correlated with another aromatic proton H_c_, which signified a four proton *meta* coupling (Fig. 4 and S26†). Moreover, proton H_b_ correlated with the low field –CH_2_ proton, signifying a four bond allylic coupling (Fig. 4 and S27†). These two observations stated above strongly confirm that one part of the ring is aromatic and the other is not, thus explaining the loss of the *C*_2_-symmetry in the ^1^H-NMR spectrum. Furthermore, these H_a_ and H_b_ protons were found to correlate in the nuclear Overhauser effect (NOE) spectrum, thus confirming the relationship between the aromatic and the non-aromatic parts (Fig. 4 and S28†). Also, H_f_ correlated to H_g_*via* the NOE, proving the connection between the photo-modified and nascent parts of the molecule (Fig. 4 and S28†). Thus, the results derived from the NMR spectra confirmed that the di-hydrophenanthrene (peak 2 in Fig. 2h) that we obtained is the 1,2-di-hydro derivative (**f_1pp_**). In addition to the above discussed photoproducts, the fractions **f_1_** and **f_2_** also contained decarboxylated products of the di-hydrophenanthrene intermediate (**f_1dc_**) and phenanthrene, respectively, as minor products, as elucidated from the chromatogram peaks at higher retention times, which have also been characterized with various techniques (Fig. S12 and S29–S33†). We hypothesize that the parent structure of **f_1pp_** and **f_1dc_** is indeed **DHPS**, and **f_1pp_** is perhaps formed by a sequence of [1,3]-sigmatropic shifts from **DHPS** (Fig. S34†). We also hypothesize that the origin of the decarboxylated derivative is an offshoot of the main reaction step. Thus, the net composition of **DHPS** formed could be approximately translated to the summation of **f_1pp_** and **f_1dc_** (Fig. S34†).

Coming back to the reaction in series scheme, the time evolution shown in Fig. 2g resembles one in which *k*_1_ > *k*_2_ (Fig. S35†). Hence, fitting the kinetics to the appropriate function, we derived the rate constants as *k*_1_ = 0.1 s^–1^ and *k*_2_ = 1.2 × 10^–2^ s^–1^ (Fig. S36†). This particular photo-activity also suggests that in clay conjugates, *k*_1_ was enhanced 100 fold compared to the nascent **TPTS** solution. The enhancement was to the extent that *k*_1_ eventually became larger than *k*_2_, hence, suggesting that the rate of formation of **DHPS** became higher than the rate of its subsequent oxidation. **DHPS**, however, is not photo-stable and owing to its photo-reactivity, is converted to **f_1pp_**, which is eventually oxidized to form **PHES** (Fig. S34†). To the best of our knowledge, the observations made above make this the only system in which the di-hydrophenanthrene derivative has been observed in aerobic conditions with a complete elucidation of the structure.

**Fig. 5:**
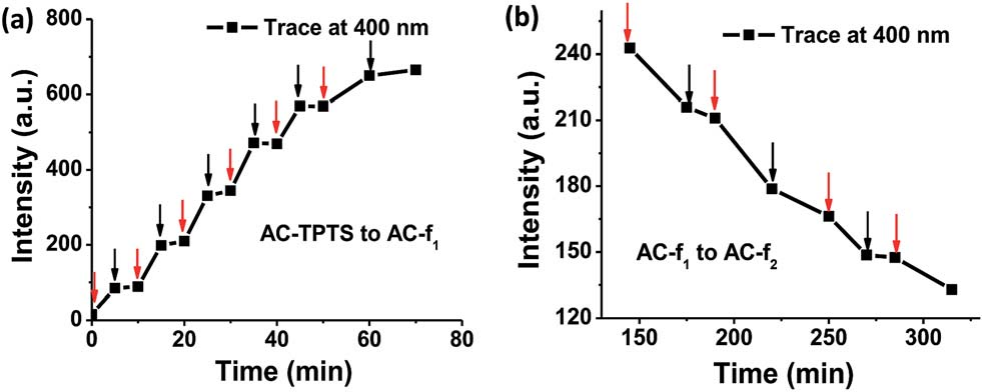
(a) and (b) show the emission intensity of **AC** (0.9 wt%) conjugated with **TPTS** (10^–4^ M) (*l* = 1 cm, *λ*_exc_ = 350 nm, water) followed at 400 nm under light (red arrows, 254 nm irradiation) and dark conditions (black arrows) for various times ((a) **AC-TPTS** to **AC-f_1_**, (b) **AC-f_1_** to **AC-f_2_**).

Di-hydrophenanthrene derivatives are known to be highly susceptible to oxidation and most times atmospheric oxygen is capable of converting them to their oxidized counterpart.^9^ Therefore, we purged a methanol solution of **f_1pp_** with oxygen gas for over 30 min. To our surprise absolutely no changes were observed in the UV/vis absorption and fluorescence measurements (Fig. S37†). This was quite anomalous as it hinted towards the fact that the oxidation of **f_1pp_** to form phenanthrene is not driven by oxygen alone, which is contrary to conventional wisdom. Fig. 5a and b show that as the 254 nm irradiation over the clay–dye conjugate (**AC-TPTS**) is switched off, the rates of both processes (**AC-TPTS** to **AC-f_1_** and **AC-f_1_** to **AC-f_2_**) are significantly retarded. This points to the fact that not only is **TPTS** to **f_1pp_** governed by light, **f_1pp_** to **PHES** is also critically photo-controlled. Since the final oxidation cannot be done by light alone, this hints that in this case **f_1pp_** to **PHES** is a rare situation of a “true” photo-oxidation, which refers to the interaction of the excited state of the molecule with oxygen to give the oxidation product.^18^ Furthermore, we investigated the possibility of propyl amine chains (which are a component of **AC**) acting as a base in the photo-reaction.^19^ Hence, we carried out the irradiation of **TPTS** in the presence of varying amounts of *n*-propyl amine (Fig. S38–S40†). The kinetics of these transformations were far too slow to be compared with **AC**, and to have any significant impact on the reaction outcome (Fig. S41 and S42†). These results clearly rule out the contribution of amino propyl groups as a base in clay for influencing the reaction rates and thus further reiterate that the conformation restriction perhaps plays a major role in the transformation.^20^,^21^

## Conclusions

**Scheme 1:**
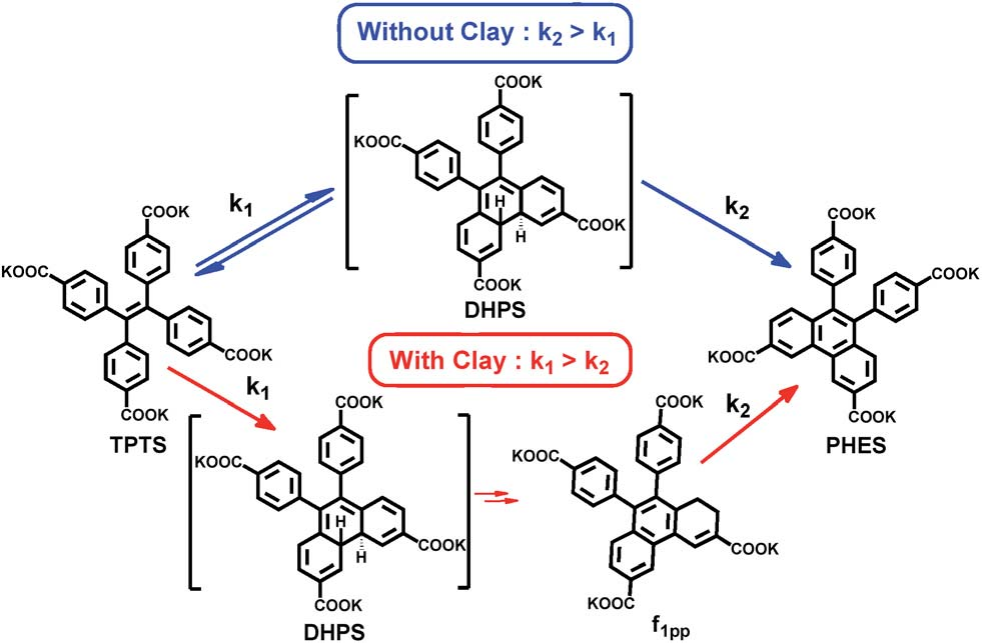
Reaction sequence for nascent **TPTS** (blue arrows) and **TPTS** inside **AC** (red arrows).

In conclusion, we have successfully shown that interlayer galleries can be used as templates to explore the increased rate of photo-oxidation reactions in stilbene-like systems. We show that there is a marked change in the rate constants of the photo-oxidation once **TPTS** is conjugated in galleries of amino clay (Scheme 1). This change points towards the idea that restricted rotation of the phenyl rings of **TPTS** on the **AC** sheet is an important factor in tipping the scales of the rate constants. On clay, the ring closure, which was initially a slow step, becomes 100 times faster, hence, overtaking the oxidation step. Even though one of the demanding features of this conjugate is that it perhaps requires a strong association of the substrates to the template, hence the use of negatively charged molecules for a positively charged template, this presents a unique case in which electrocyclic ring closure is modulated on clay surfaces. This system also presents a unique study where the usually unstable, di-hydrophenanthrene derivative was isolated in the form of 1,2-di-hydrophenanthrene and structurally analysed, giving a major insight into the reaction process. The study thus opens the door to investigations of reactions involving a variety of chromophores on layered materials as well as other varieties of organic–inorganic hybrids, and not just for their rates of reactions but also for the stabilization of unusual intermediate states, which can be achieved by this strategy.^22^

## Supplementary Material

Supplementary information

## References

[cit1] Hanoian P., Liu C. T., Hammes-Schiffer S., Benkovic S. (2015). Acc. Chem. Res..

[cit2] Kawamichi T., Haneda T., Kawano M., Fujita M. (2009). Nature.

[cit3] Kang J., Rebek Jr J. (1997). Nature.

[cit4] Thomas J. K. (1988). Acc. Chem. Res..

[cit5] Takagi K., Shichi T., Usami H., Sawaki Y. (1993). J. Am. Chem. Soc..

[cit6] Wang Q., Mynar J. L., Yoshida M., Lee E., Lee M., Okuro K., Kinbara K., Aida T. (2010). Nature.

[cit7] Ohkubo K., Nanjo T., Fukuzumi S. (2005). Org. Lett..

[cit8] Amaya T., Ito T., Hirao T. (2015). Angew. Chem., Int. Ed..

[cit9] Rodier J.-M., Myers A. B. (1993). J. Am. Chem. Soc..

[cit10] Lewis F. D., Kurth T. L., Kalgutkar R. S. (2001). Chem. Commun..

[cit11] Jørgensen K. B. (2010). Molecules.

[cit12] Patil A., Eswaramoorthy M., Mann S. (2004). Angew. Chem., Int. Ed..

[cit13] See ESI for HRMS and Prep HPLC

[cit14] Barbara P. F., Rand S. D., Rentzepis P. M. (1981). J. Am. Chem. Soc..

[cit15] Hong Y., Lama J. W. Y., Tang B. Z. (2009). Chem. Commun..

[cit16] Shustova N. B., McCarthy B. D., Dincă M. (2011). J. Am. Chem. Soc..

[cit17] Shustova N. B., Ong T.-C., Cozzolino A. F., Michaelis V. K., Griffin R. G., Dincă M. (2012). J. Am. Chem. Soc..

[cit18] Knittel-Wismonsky T., Fischer G., Fischer E. (1972). Tetrahedron Lett..

[cit19] Somers J. B. M., Couture A., Lablache-Combier A., Laarhoven W. H. (1985). J. Am. Chem. Soc..

[cit20] Although irradiation of TPE derivatives have been attempted in the aggregated state (ref. 9*b* and *f*), it does not yield the intermediate but rather just the phenanthrene derivative. The molecule used is substituted with electron rich moieties and thus its oxidation rates would be significantly higher compared to ones with deactivating substituents such as in the present case

[cit21] An interesting result was published as this manuscript was being prepared; He Z. Shan L. Mei J. Wang H. Lam J. W. Y. Sung H. H.-Y. Williams I. D. Gu X. Miao Q. Tang B. Z., Chem. Sci., 2015, 6 , 3538 , . This paper describes the formation of di-hydrophenanthrene derivative upon aggregation and the stabilization has been pointed towards the molecular design with more π rings. Thus the stabilization of intermediate and subsequent oxidation perhaps has some bearing on the electronic effects as a result of functional groups present in the molecule as well .28717460

[cit22] Liu M., Ishida Y., Ebina Y., Sasaki T., Aida T. (2013). Nat. Commun..

