# Supplementary material for: Shining light on clay–chromophore hybrids: layered templates for accelerated ring closure photo-oxidation
Source: Chem Sci. 2015 Jul 17;6(11):6334–40. doi: 10.1039/c5sc02215k (PMC6054095; doi:10.1039/c5sc02215k)
Supplement: Supplementary file 1 [file SC-006-C5SC02215K-s001.pdf]

Electronic Supplementary Material (ESI) for Chemical Science.
This journal is © The Royal Society of Chemistry 2015

Supporting Information

Shining the Light on Clay-Chromophore Hybrids: Layered Templates for Accelerated Ring Closure Photo-Oxidation

Ankit Jain,[†] Amritroop Achari,[‡] Nivin Mothi,[‡] Muthuswamy Eswaramoorthy*[†‡] and Subi J. George*[†]

[†]New Chemistry Unit, Jawaharalal Nehru Centre for Advanced Scientific Research (JNCASR), Bangalore, India

[‡]Chemistry and Physics Materials Unit, JNCASR, Bangalore, India

E-mail: george@jncasr.ac.in, eswar@jncasr.ac.in; Fax: +91 80 22082627; Tel: +91 80 22082964;

Table of Contents:

A. General Methods

B. Synthesis

C. Supporting Figures

A. General Methods. Unless otherwise noted, all reagents were used as received and all reactions were carried out under an argon atmosphere. All NMR experiments were recorded on Bruker AVANCE-400 MHz spectrometer at room temperature.

1. **Optical spectroscopy:** UV/Vis absorption and fluorescence spectra were recorded on a Perkin-Elmer Lambda 750 and Perkin-Elmer LS 55 spectrometer respectively. ATR-IR was measured on Perkin Elmer frontier machine with Pike gladiatr and diamond crystal. Range of measurement was from 400 to 4000 cm^{-1}, data interval was 0.2 cm^{-1}, resolution was 4 cm^{-1} and 16 scans were collected.

2. **Irradiation:** Irradiation was done in a 10 mm quartz cuvette (for small scale) and in a 100 ml glass beaker (for large scale). Lamp used was OSRAM puritec germicidal lamp HNS, 8W, G5 (G8T5/OF). In a general procedure the solution was placed inside the lamp chamber directly under the lamp.

3. **ESI-HRMS:** High Resolution Mass Spectra (HRMS) were recorded on a Agilent 6538 Ultra High Definition (UHD) Accurate-Mass Q-TOF-LC/MS system using electrospray ionization (ESI) (negative mode). Liquid chromatography (LC) is coupled with the instrument. Column used is Agilent ZORBAX eclipse plus C18 rapid resolution. Injection volume is 10ul methanol solution, eluent gradient is 40% MeCN to 90% MeCN over 40 mins. The other part of the solvent composition was 1% Ammonium formate in water. Flow rate is 0.4 ml/min. Chromatograms from Figures 2d and S3a are from this method.

4. **Preprative HPLC:** Shimadzu LC-8A with phenomenex luna 10u C18 (2), 250 X 10 mm, 10 micron was employed. Injection volume was 1 ml methanol solution, eluent gradient was 40% MeCN to 60% MeCN over 30 mins. The other part of the solvent composition was water. 0.1% Triflouro acetic acid was used in all eluents. Flow rate was 8 ml/min. Chromatograms from Figures 2h and S12 are from this method.

   ***[13]** Chromatograms from figures 2d and 2h probe the same species but the retention times are evidently different this is because for figure 2d HPLC column mentioned in ESI-HRMS was used and for 2h the one mentioned in preparative HPLC was employed. The reason behind this choice is that after clay extraction the methanol solution could still be not used in the ESI-HRMS as even very low concentration of residual clay saturated the MS chromatogram. Therefore in case of clay extracts a preparative HPLC was done (chromatograms of which are in figure 2h) and after purification of each fraction MS was performed individually. MS and the UV absorption spectra corroborated with the fact that we are monitoring the same species in figure 2d and 2h.

5. **Dynamic Light scattering and Zeta potential measurement:** Measurements were carried out using a NanoZS (Malvern, UK) employing a 532 nm laser at a back scattering angle of 173°.

B. Synthesis. **TPTS** was prepared according to the reported procedure [16]. ^{1}H-NMR, ^{13}C-NMR, HPLC and HRMS have been shown in Figures S1-S3. **TPTS acid**: ^{1}H-NMR (400 MHz, D_2O): δ = 7.80 (d, J = 8.4 Hz, 8H), 7.13 (d, J = 8.4 Hz, 8H)

^{13}C-NMR (100 MHz, D_2O): δ = 175.20, 145.91, 141.30, 134.53, 130.95, 128.46

HRMS (ESI, negative mode) *m/z* (%): 507.1975 $[M-1]^-$, calculated for $C_{30}H_{20}O_8$, 508.1158 $gmol^{-1}$

C. Supporting Figures.

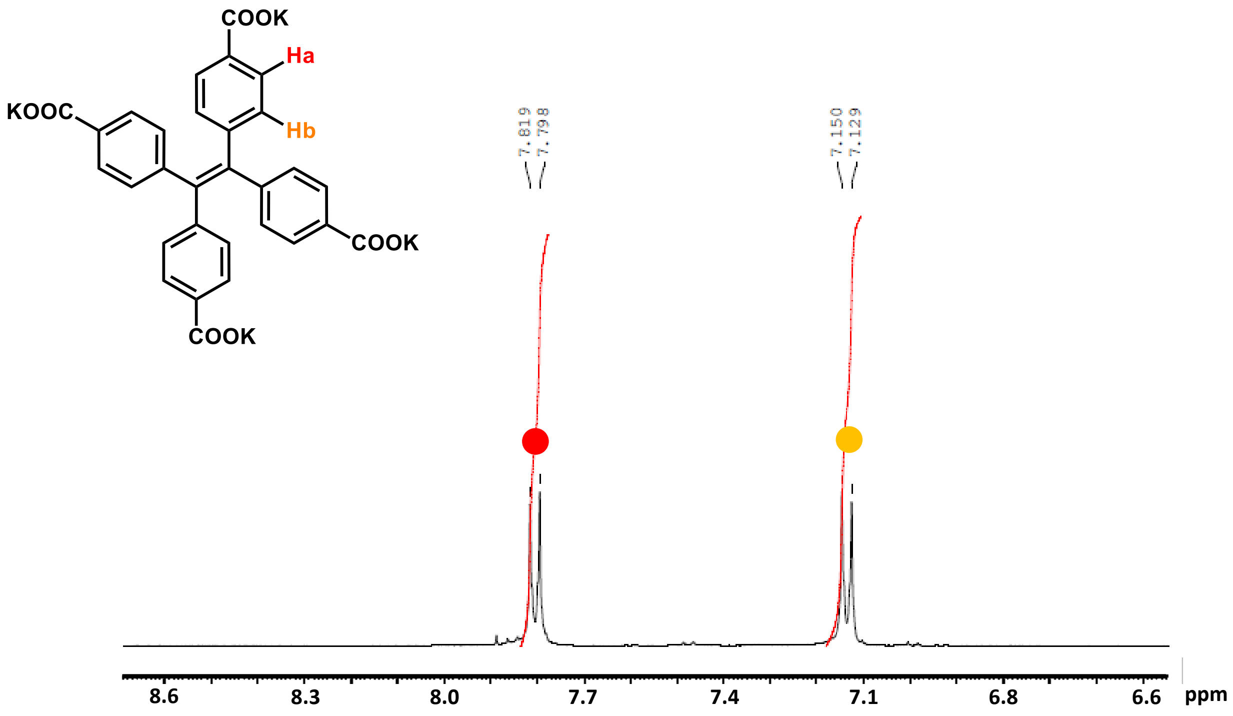

Figure S1. ^{1}H-NMR spectrum of **TPTS** in D_2O.

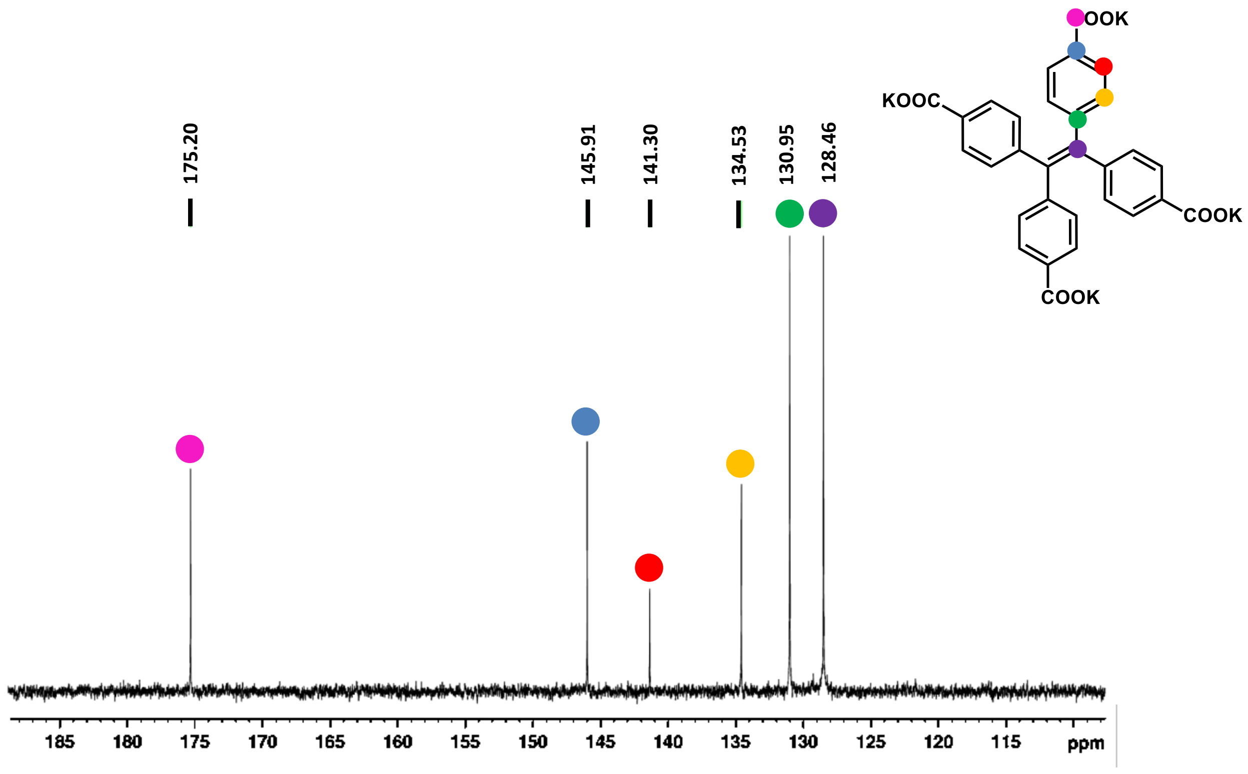

Figure S2. ^{13}C-NMR spectrum of **TPTS** in D_2O.

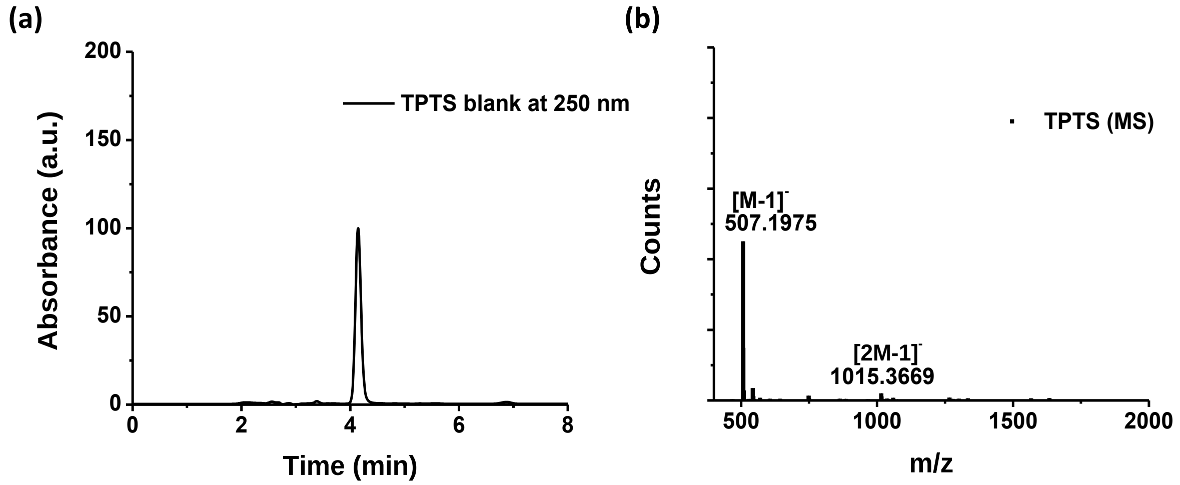

Figure S3. a) HPLC trace of **TPTS acid** obtained by monitoring the absorbance at 250 nm, b) ESI-HRMS of **TPTS acid.**

***Explanation*:** Figure S3b shows the ESI-MS of **TPTS acid** in negative ion mode, hence showing the [M-1] peak. Interestingly, molecule also shows a [2M-1] peak which corresponds to H-bonded acid-dimer formed in MS conditions. H-bonded dimers of carboxylic acids in ESI-MS are very well known in literature.

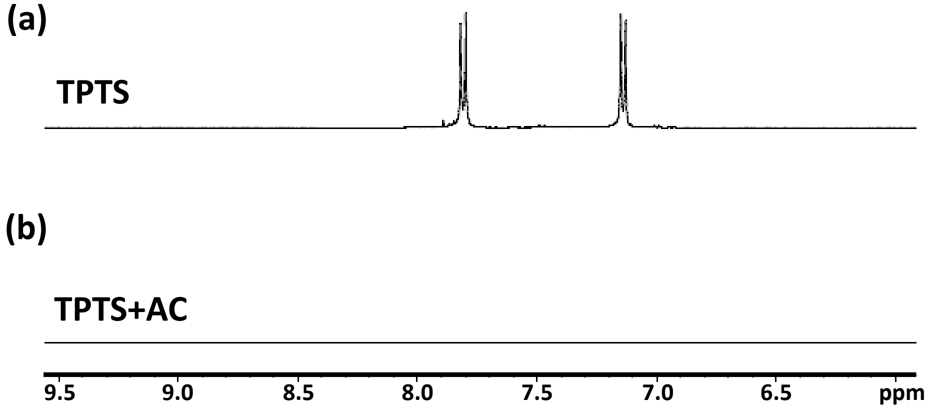

Figure S4. a) and b) ^{1}H NMR spectra of **TPTS** in D_2O and **TPTS+AC** conjugate respectively

Explanation: Figure S4 shows that as soon as **TPTS** is conjugated to 0.9 wt% **AC** the phenyl proton peaks disappear in the conjugate. We hypothesize this is due to the increase in T_2 relaxation time caused by the restricted rotation of phenyl rings on clay surfaces. This increase in T_2 is then responsible of broadening to the extent of flattening the proton peaks.

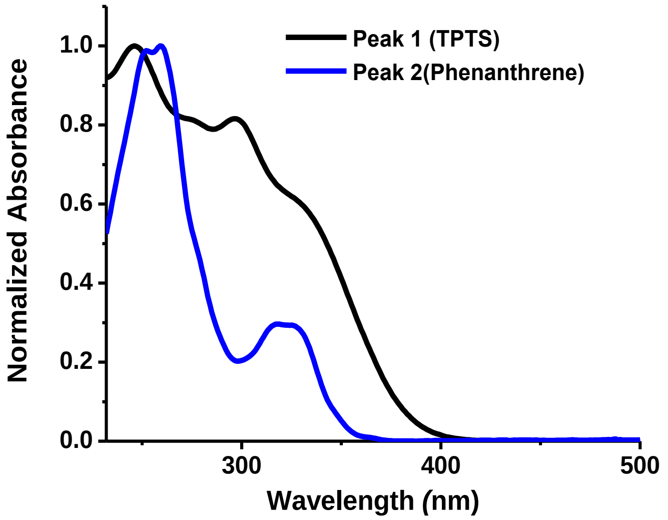

*Figure S5.*UV absorption spectra of respective HPLC peaks in Figure 2(d) in the main text.

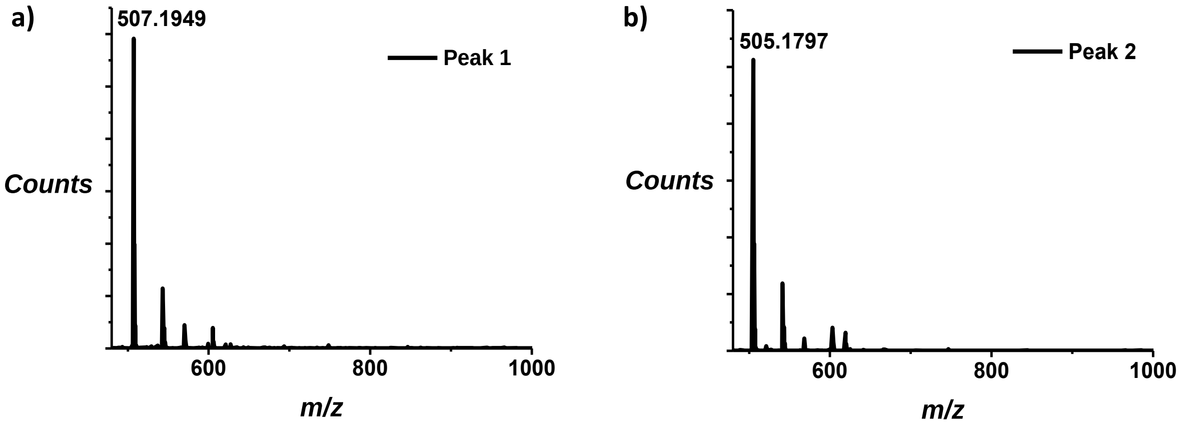

Figure S6. ESI-HRMS of respective HPLC peaks in Figure 2(d) in the main text.

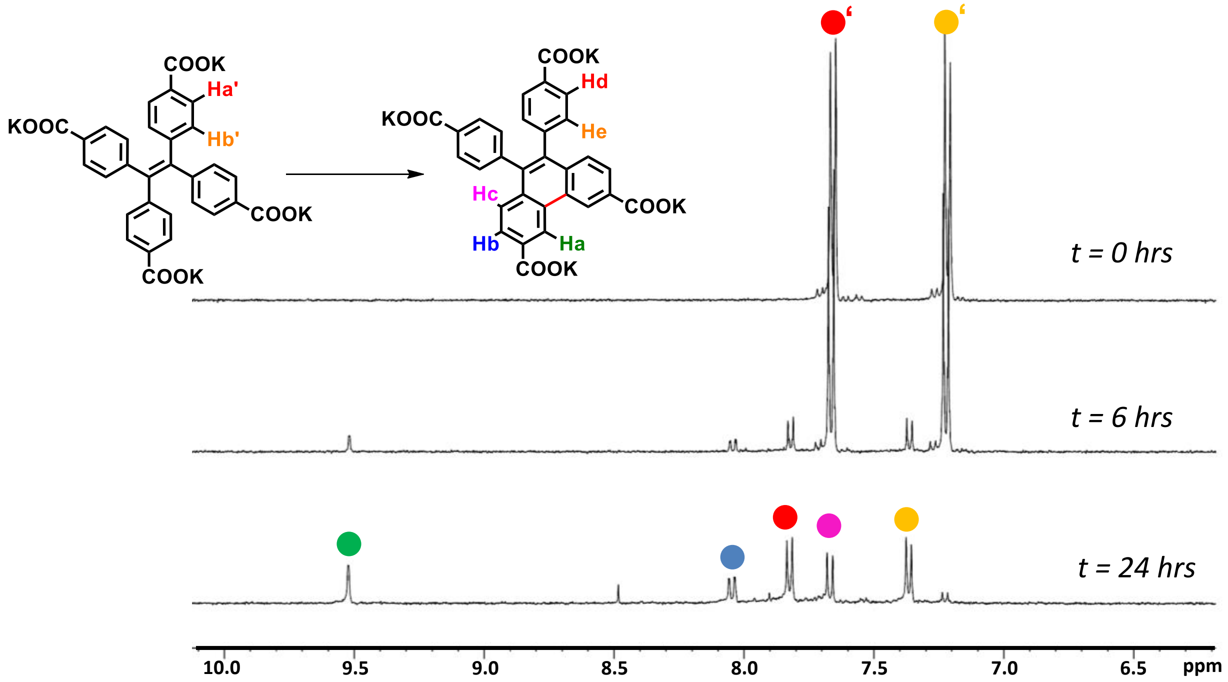

Figure S7. ^{1}H-NMR spectra of **TPTS** in D_2O under various times of 254 nm illumination, inset shows the molecular structure of the transformation.

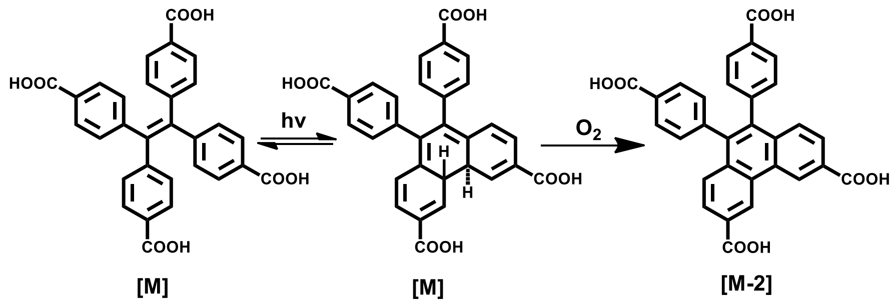

$$A \xrightarrow{k_1} B \xrightarrow{k_2} C$$

$$\frac{d[A]}{dt} = -k_1[A] \quad \text{......(1)}$$

$$\frac{d[B]}{dt} = k_1[A] - k_2[B] \quad \text{......(2)}$$

$$\frac{d[C]}{dt} = k_2[B] \quad \text{......(3)}$$

$$[A] = [A]_o\, exp(-k_1 t) \quad \text{......(4)}$$

$$[B] = \frac{k_1}{k_2 - k_1}\{exp(-k_1 t) - exp(-k_2 t)\}[A]_o \quad \text{......(5)}$$

$$[C] = \left(1 + \frac{k_1 exp(-k_2 t) - k_2 exp(-k_1 t)}{k_2 - k_1}\right)[A]_o \quad \text{......(6)}$$

$$At\; k_2 >> k_1$$
$$[C] = [A]_o(1 - exp(-k_1 t)) \quad \text{......(7)}$$

Figure S8. Kinetic equations showing the expression for a series reaction approximated for conditions of $k_2 >> k_1$.[a]

[a] Arnaut, L.; Formosinho, S.; Burrows, H. in *Chemical Kinetics From Molecular Structure to Chemical Reactivity*, *Ed. 1*, Elsevier Science, **2006**, pp. 86-87.

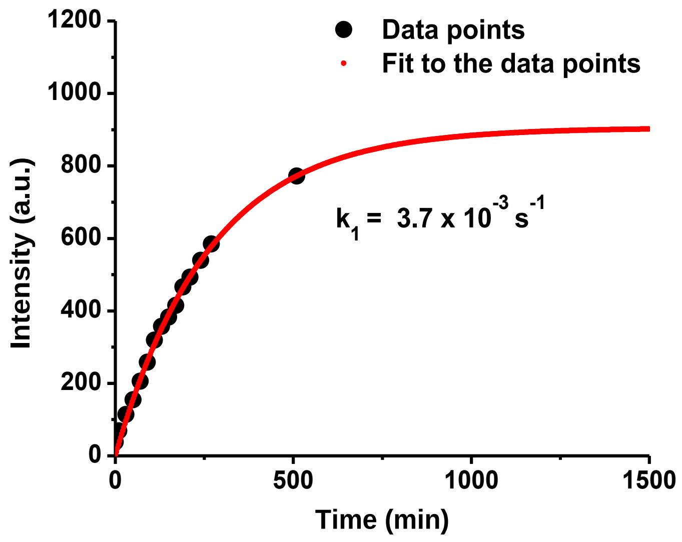

Figure S9. Emission intensity trace at 400 nm of the photo-oxidation profile of **TPTS** (*10^{-4}M, l = 1 cm, λ_{exc} = 350 nm, water*) under 254 nm irradiation with a fit to equation 7 shown in Figure S8.

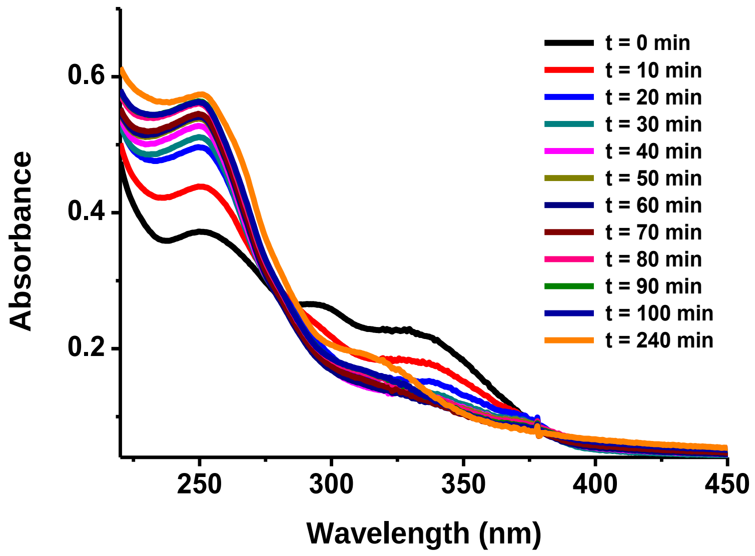

Figure S10. Time dependent UV absorption spectra of **TPTS** and **AC** conjugate (*10^{-4}M + 0.9 wt%, l = 1 cm, water*) under 254 nm illumination.

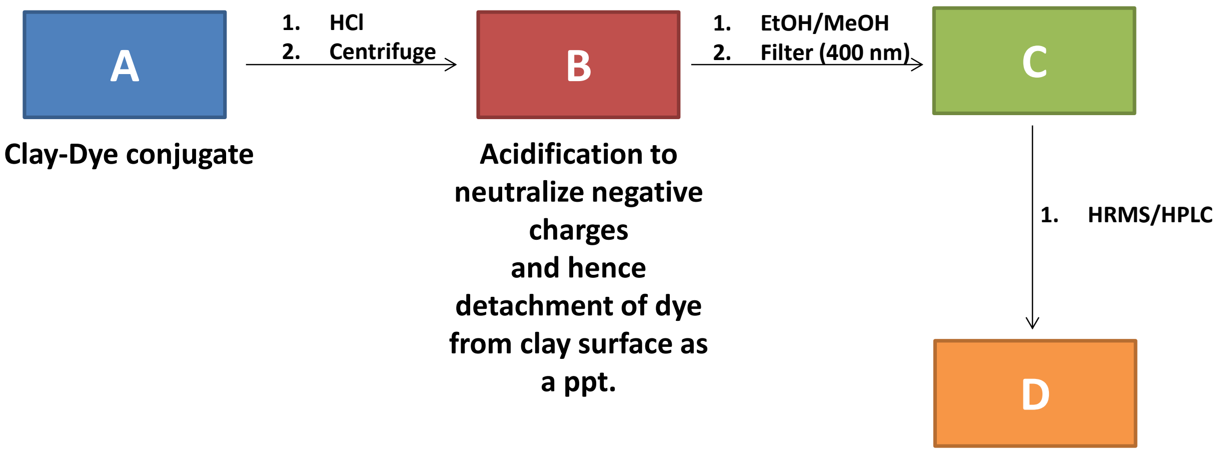

Figure S11. Extraction procedure of **TPTS** from clay.

Explanation: HRMS/HPLC carried out on the acidified product i.e. the carboxylic acid and not the carboxylate

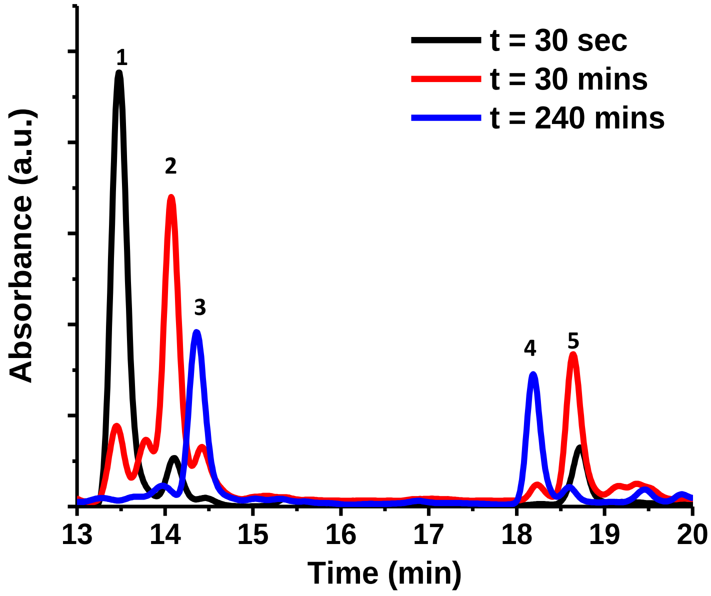

*Figure S12.*Complete HPLC profile of Figure 2(h) in the main text.

***Explanation*:** Figure S12 shows the complete HPLC profile of **TPTS acid** extracts from **AC** at various intervals. Peaks numbered 1,2 and 3 are same as one shown in Figure 2(h) in the main text. Peaks 4 and 5 are the by-products during the photo-irradiation. They are primarily decarboxylated versions of **2** and **3** (*vide infra*).

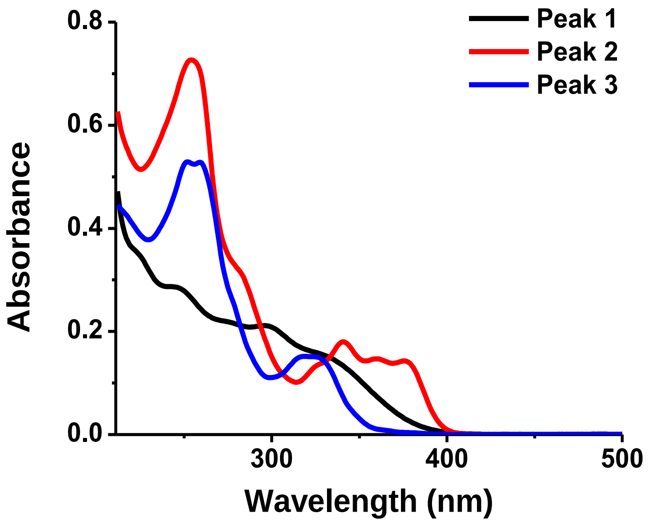

Figure S13. UV absorption spectra of respective HPLC peaks in Figure 2(h)

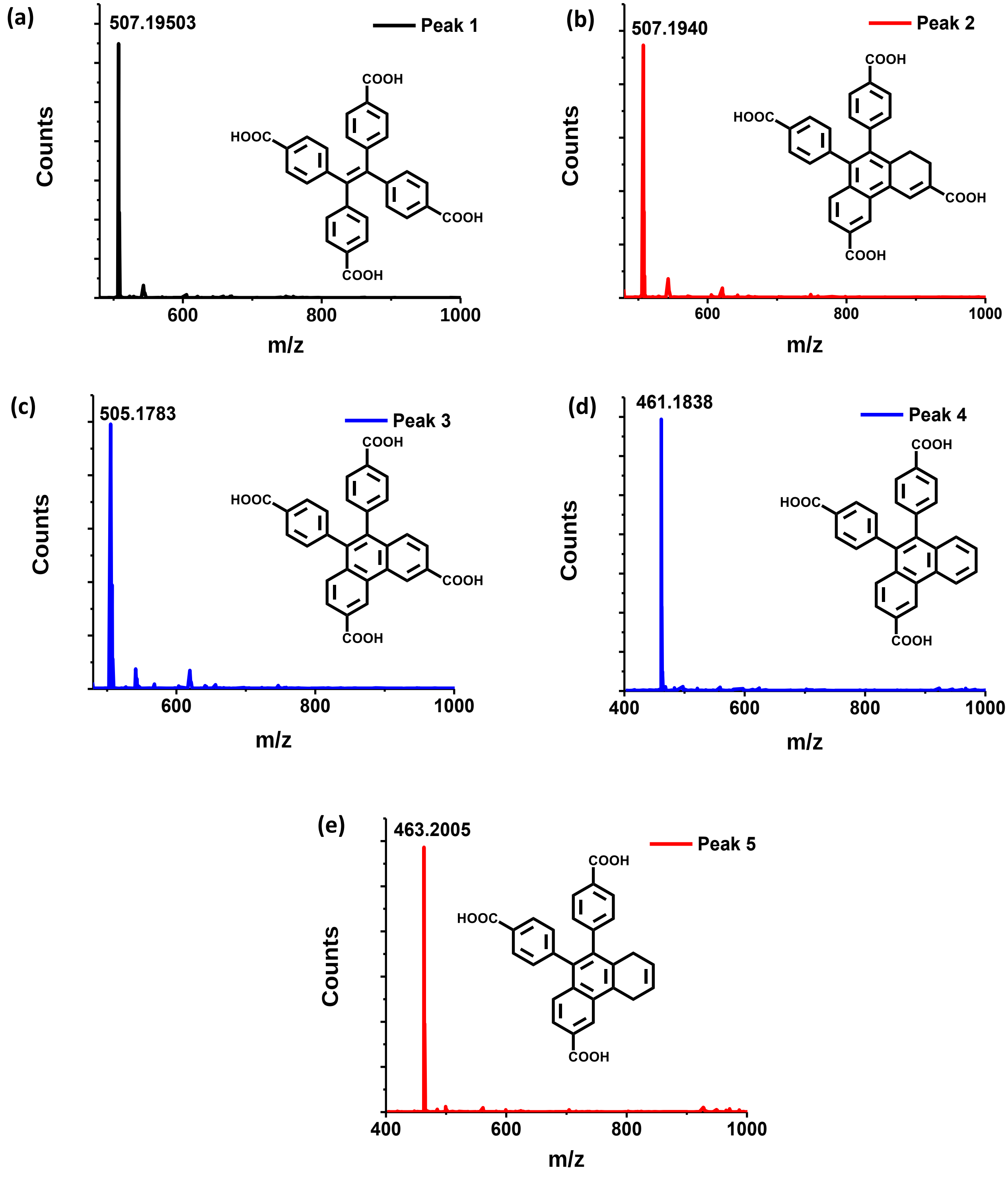

Figure S14. ESI-HRMS of respective HPLC peaks in Figure 2(h) and Figure S12.

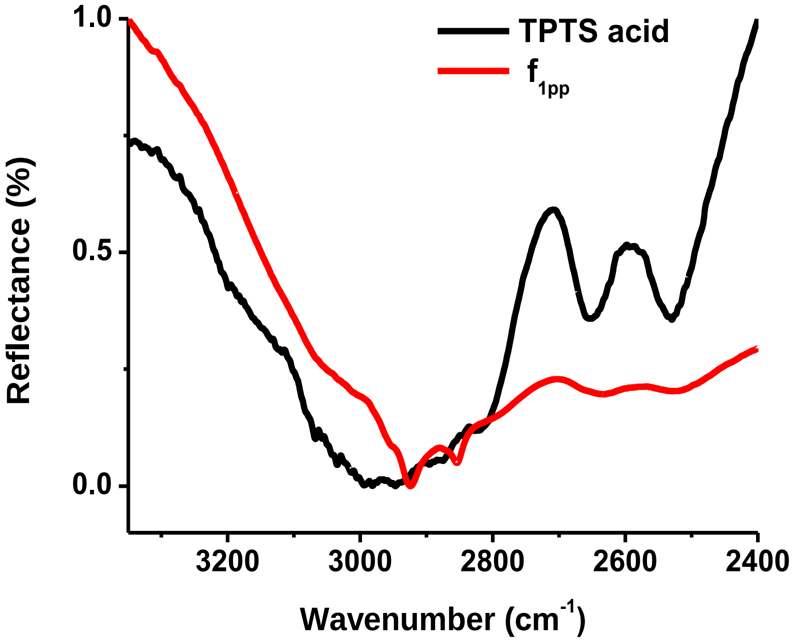

Figure S15. ATR-IR spectra of **TPTS acid** and extracted $\mathbf{f_{1pp}}$.

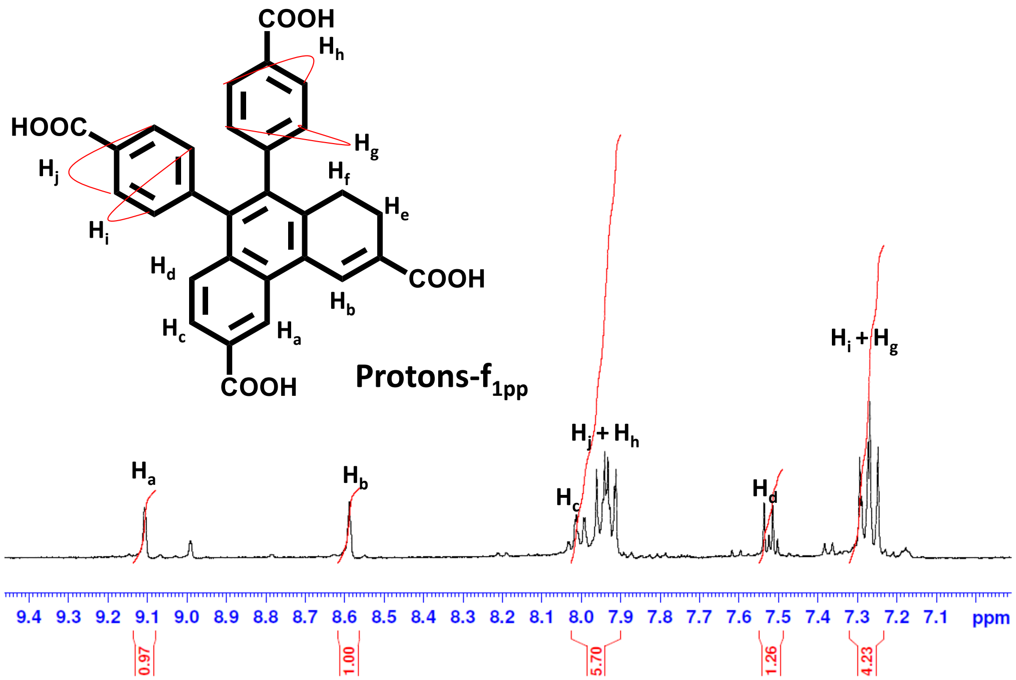

Figure S16. ^{1}H-NMR of $\mathbf{f_{1pp}}$ in CD_3OD as shown in Figure 4 with corresponding integrals (From region 7-9.5 ppm)

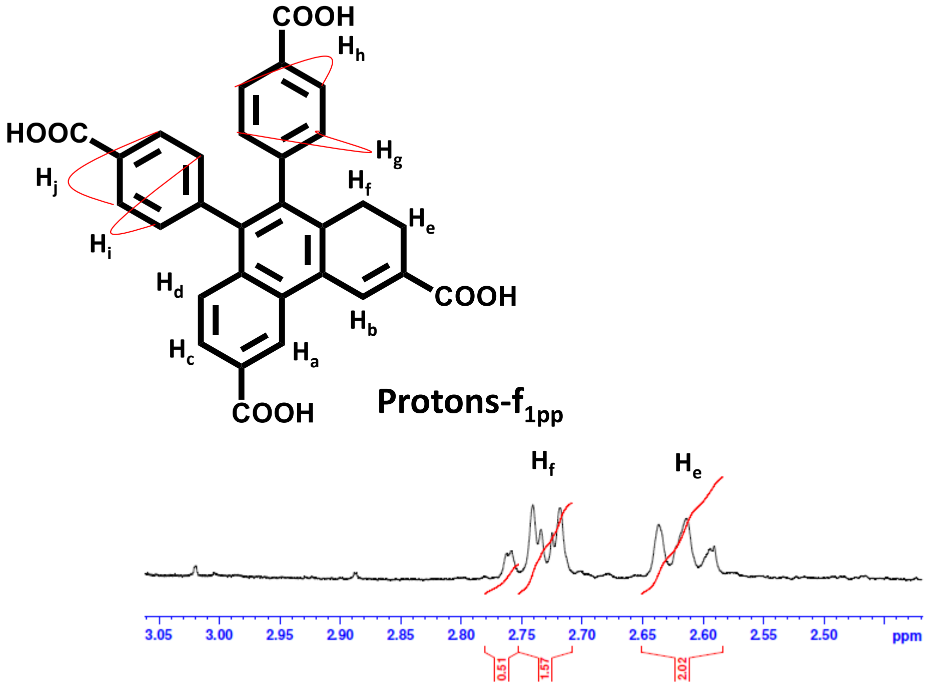

Figure S17. ^{1}H-NMR of $\mathbf{f_{1pp}}$ in CD_3OD as shown in Figure 4 with corresponding integrals (From region 2.5-3.0 ppm)

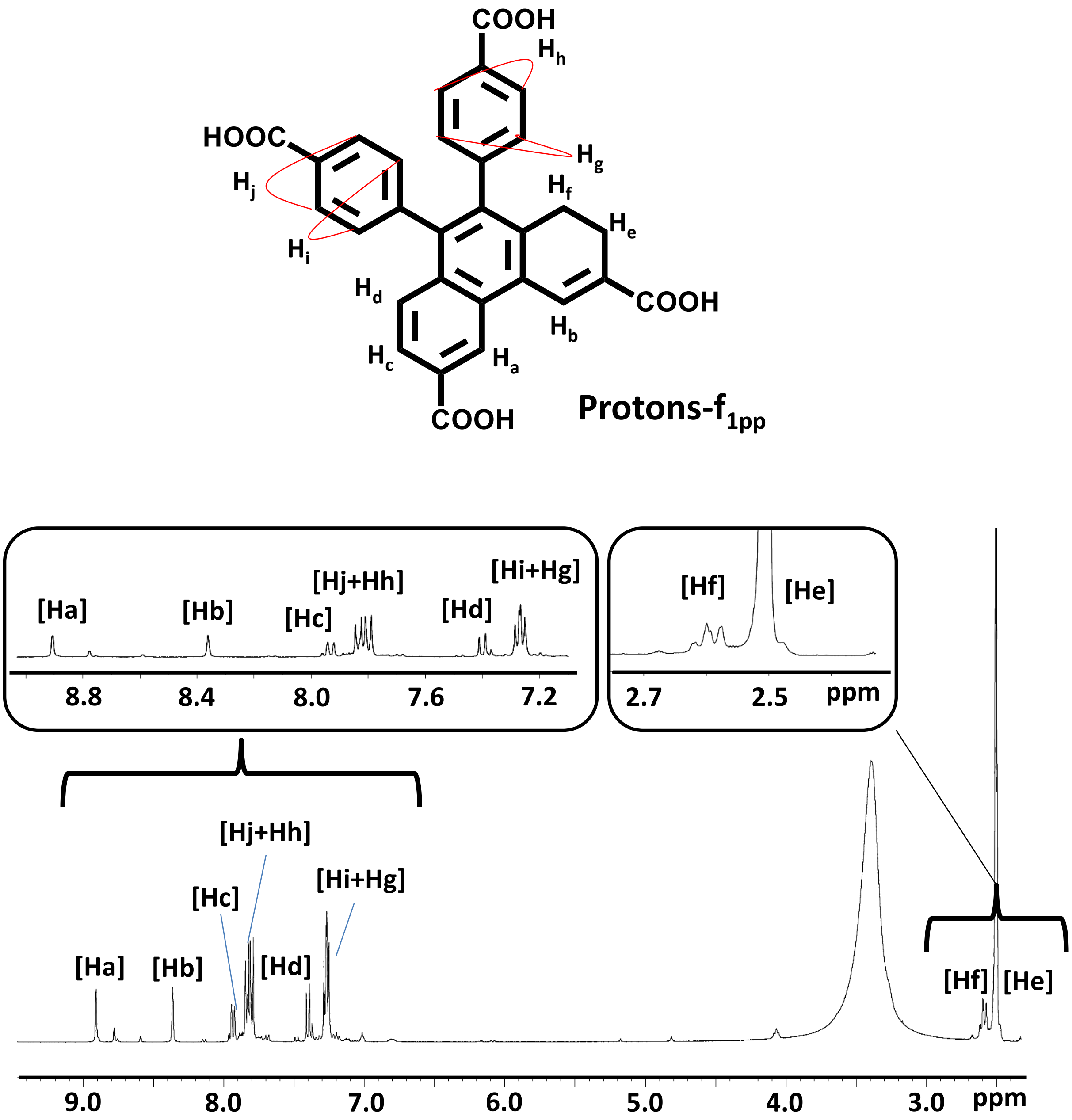

Figure S18. ^{1}H-NMR spectra of $\mathbf{f_{1pp}}$ in DMSO-d_6

Explanation: Figure S18 shows the ^{1}H-NMR of $\mathbf{f_{1pp}}$ in DMSO-d_6. $\mathbf{f_{1pp}}$ is not soluble enough in CD_3OD to carry out correlation experiments hence DMSO-d_6 was used as a solvent even though DMSO peak overlaps with proton peak of H_e. Correlations pertaining to H_e can be clearly seen despite the solvent overlap (*vide infra*, correlations are not due to DMSO solvent but entirely due to H_e as evident from HETCOR spectra Figure S23).

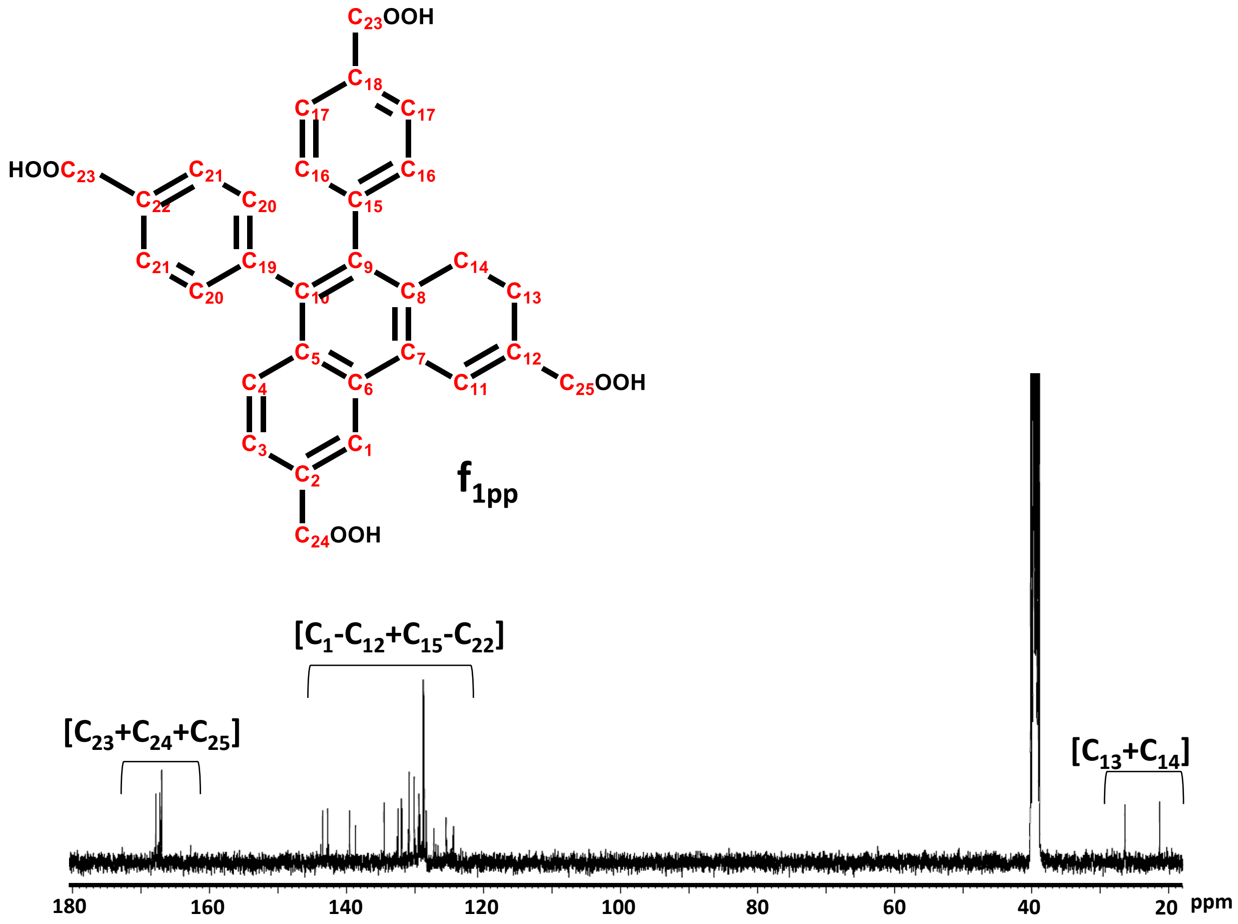

Figure S19. ^{13}C-NMR spectra of $\mathbf{f_{1pp}}$ in DMSO-d_6

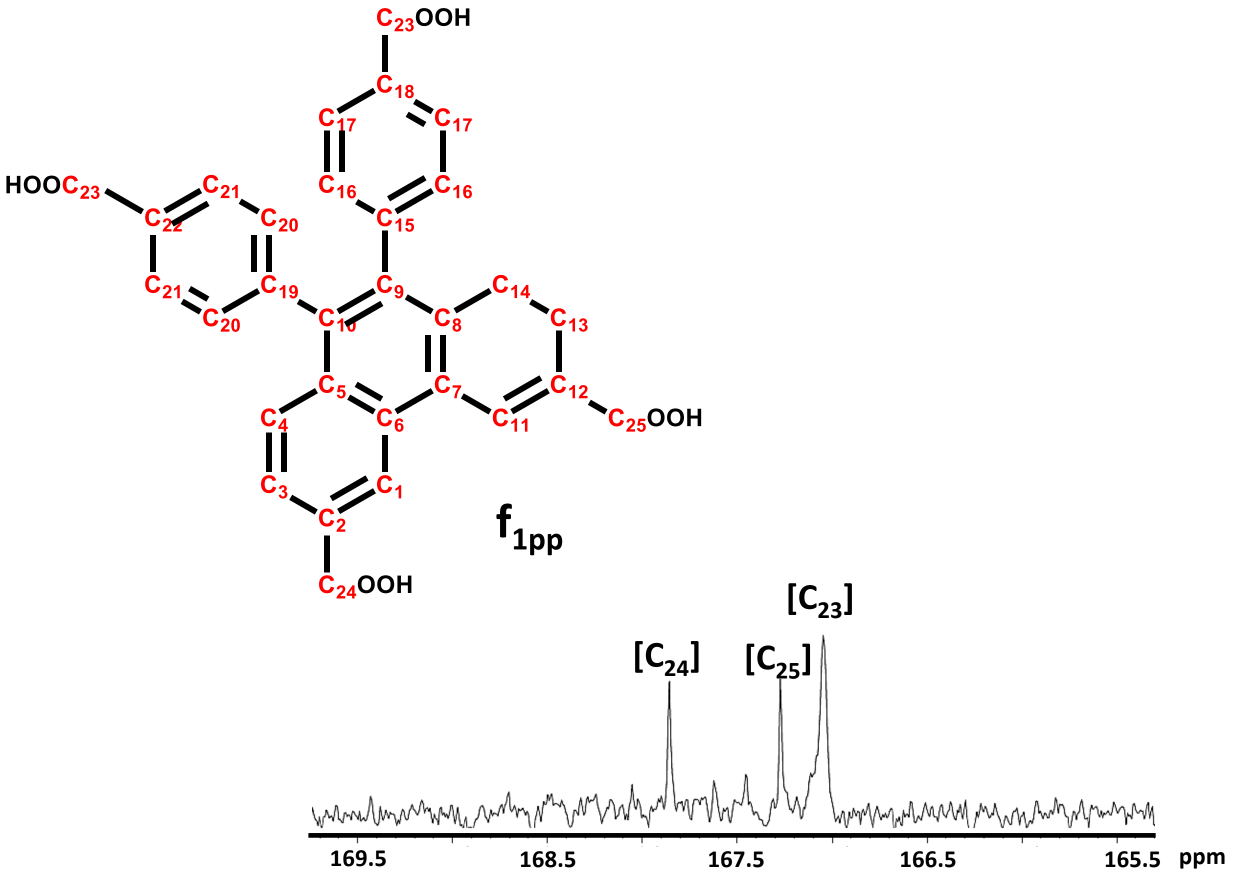

Figure S20. ^{13}C-NMR spectra of $\mathbf{f_{1pp}}$ in DMSO-d_6 zoomed in region between 165-170 ppm, 3 kinds of COOH carbons are visible which can be analyzed as: 2xC_{23}, C_{24} and $C_{25,}$ total 4 carbons.

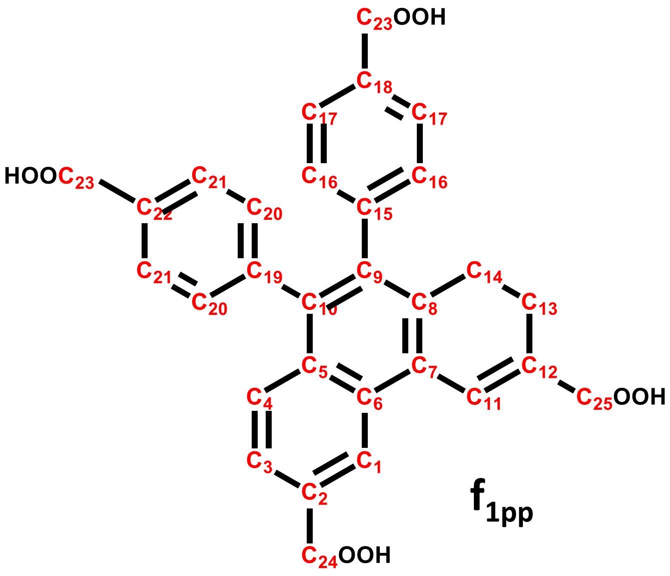

f_{1pp}

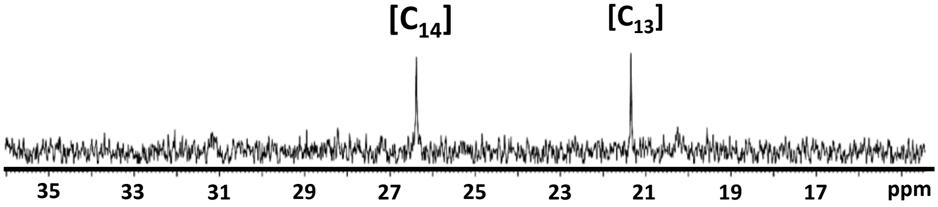

Figure S21. ^{13}C-NMR spectra of $\mathbf{f_{1pp}}$ in DMSO-d$_6$ zoomed in region between 15-35 ppm, 2 carbons in lower field suggesting two methylene carbons.

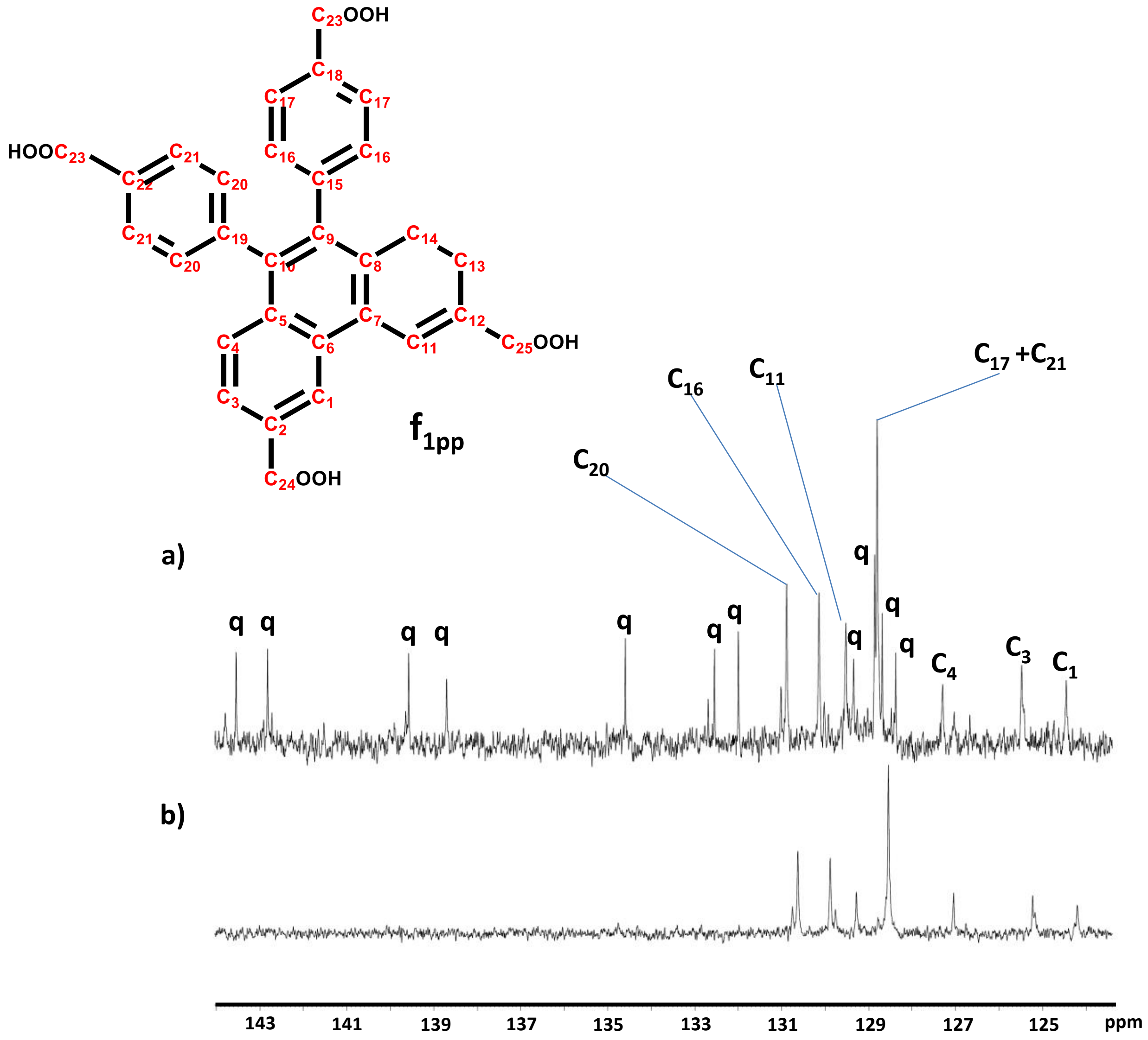

Figure S22. a) and b) ^{13}C and ^{13}C –DEPT NMR spectra respectively of $\mathbf{f_{1pp}}$ in DMSO-d_6 (peaks marked as “q” are quaternary carbons, CH carbons have been designated)

Explanation**:** Hence total **16 quaternary Carbons** (12+4); C_{18} = C_{23} (from Figure S20 and S22). Total **12 C-H Carbons** {2x(C_{16}+C_{17}+C_{20}+C_{21}) +C_{11}+ C_1+ C_3+ C_4} (from Figure S22). Total **2 CH_2 Carbons** (from Figure S21), **total 30 Carbons**

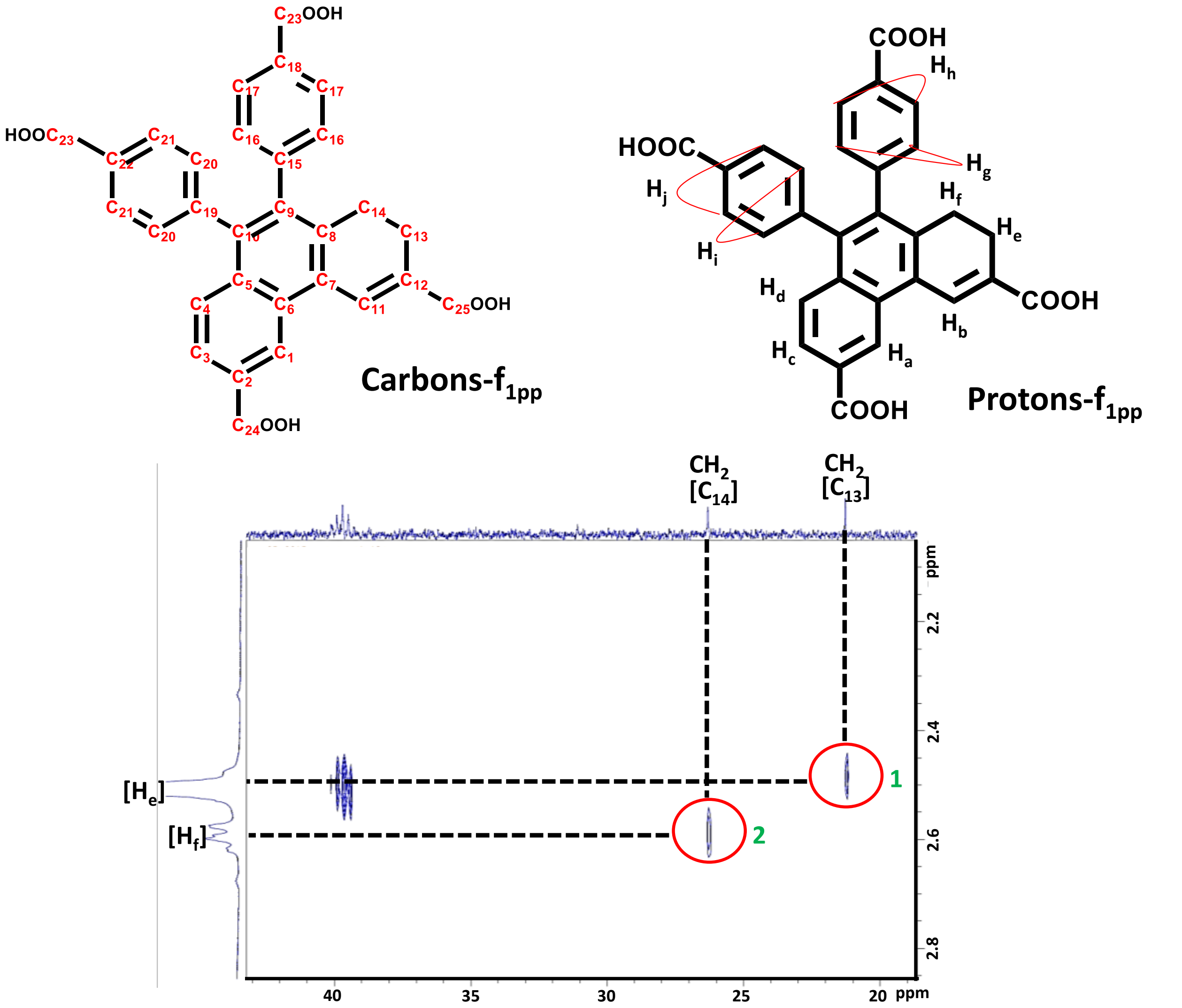

Figure S23. HETCOR 2D-NMR spectra (upfield region) showing correlation between ^{13}C and ^{1}H-nuclei of $\mathbf{f_{1pp}}$ in DMSO-d$_6$ (contours have been circled in red with correlation in black dashed lines)

Explanation: CH_2 carbons coupled to H_e and H_f

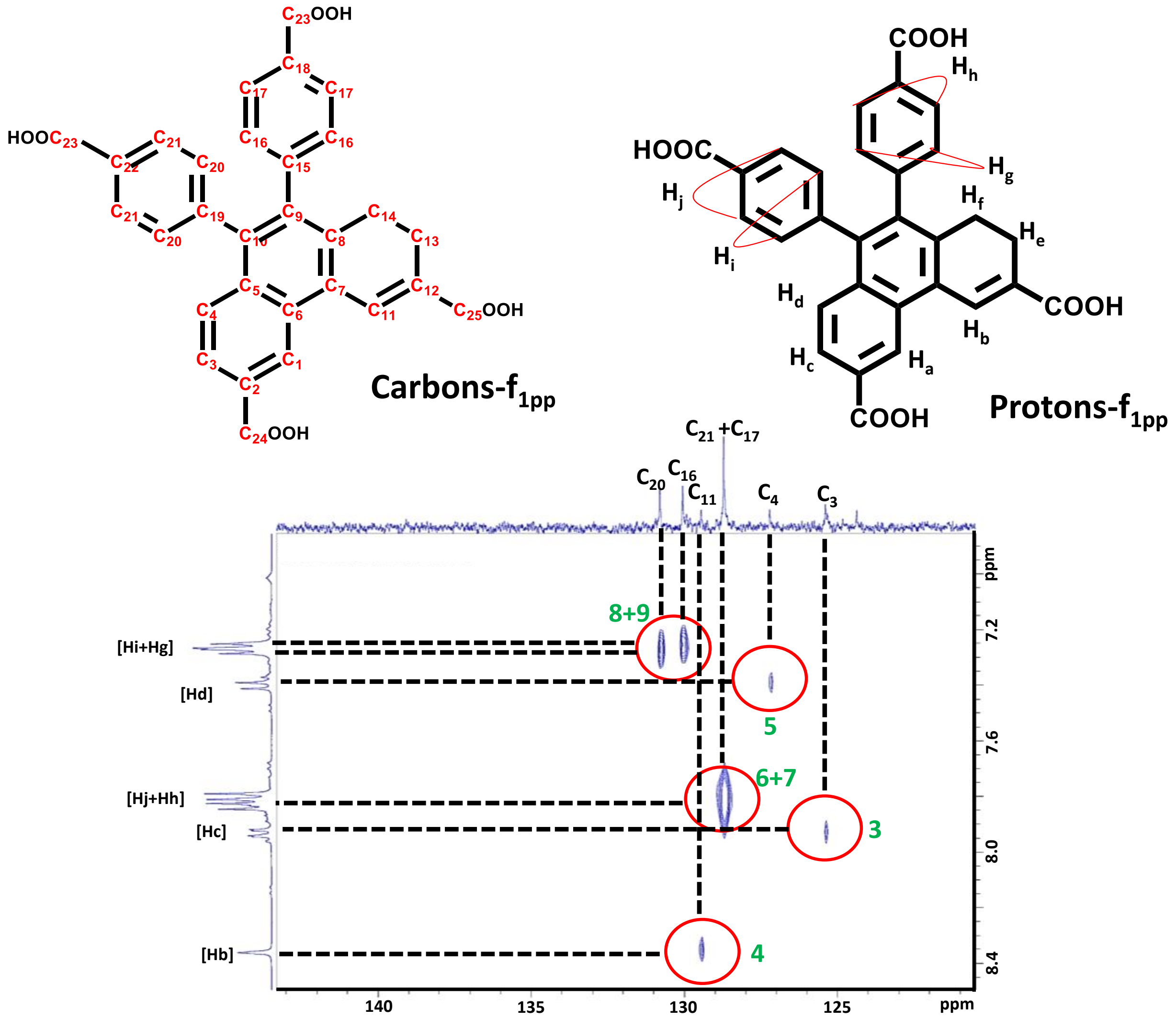

Figure S24. HETCOR 2D-NMR spectra (downfield region) showing correlation between ^{13}C and ^{1}H-nuclei of $\mathbf{f_{1pp}}$ in DMSO-d_6 (contours have been circled in red with correlation in black dashed lines).

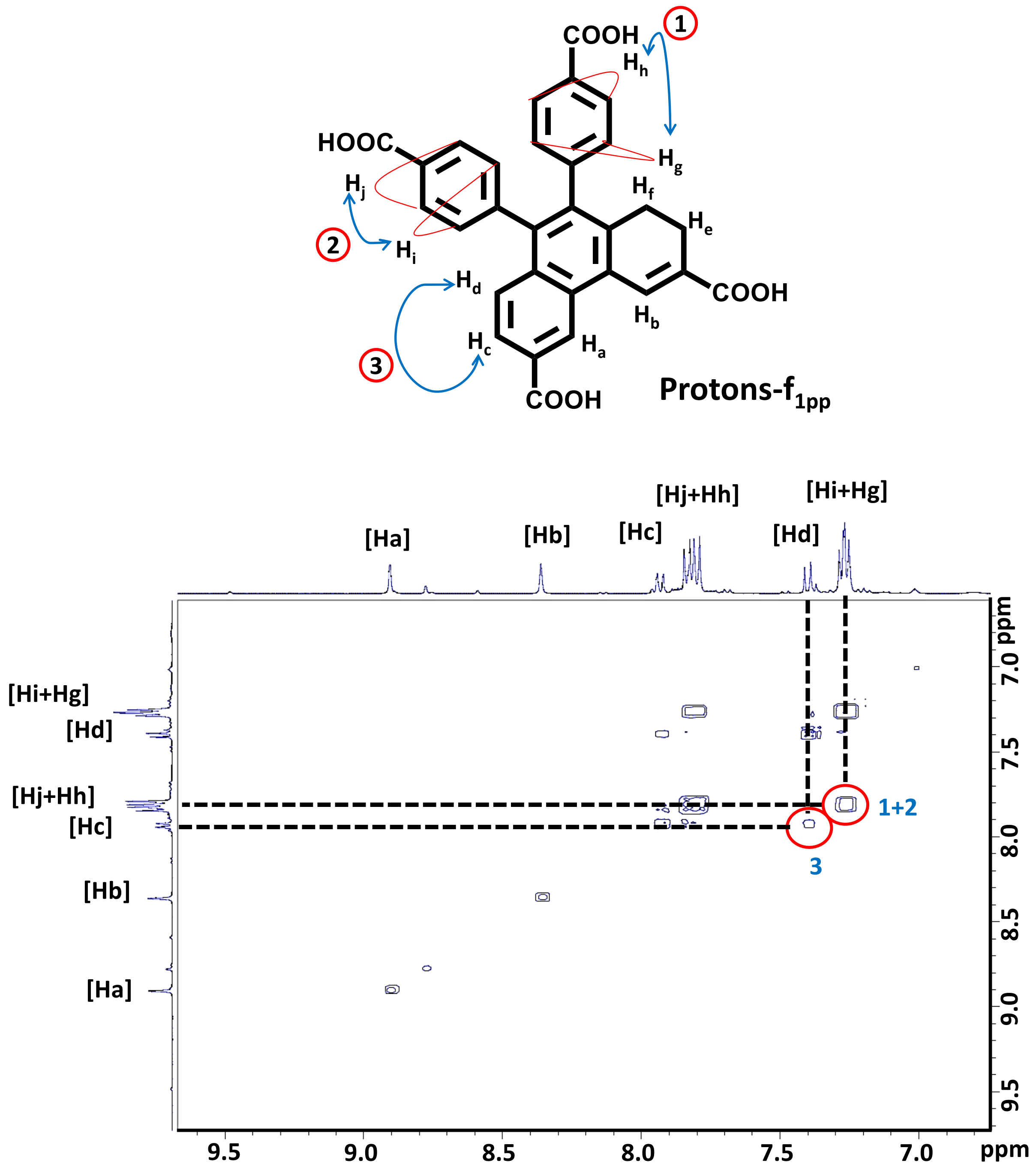

Figure S25. COSY 2D-NMR spectra showing correlation between ^{1}H-nuclei of $\mathbf{f_{1pp}}$ in DMSO-d_6 (contours have been circled in red with correlation in black dashed lines)

Explanation: 3 bond couplings in downfield region

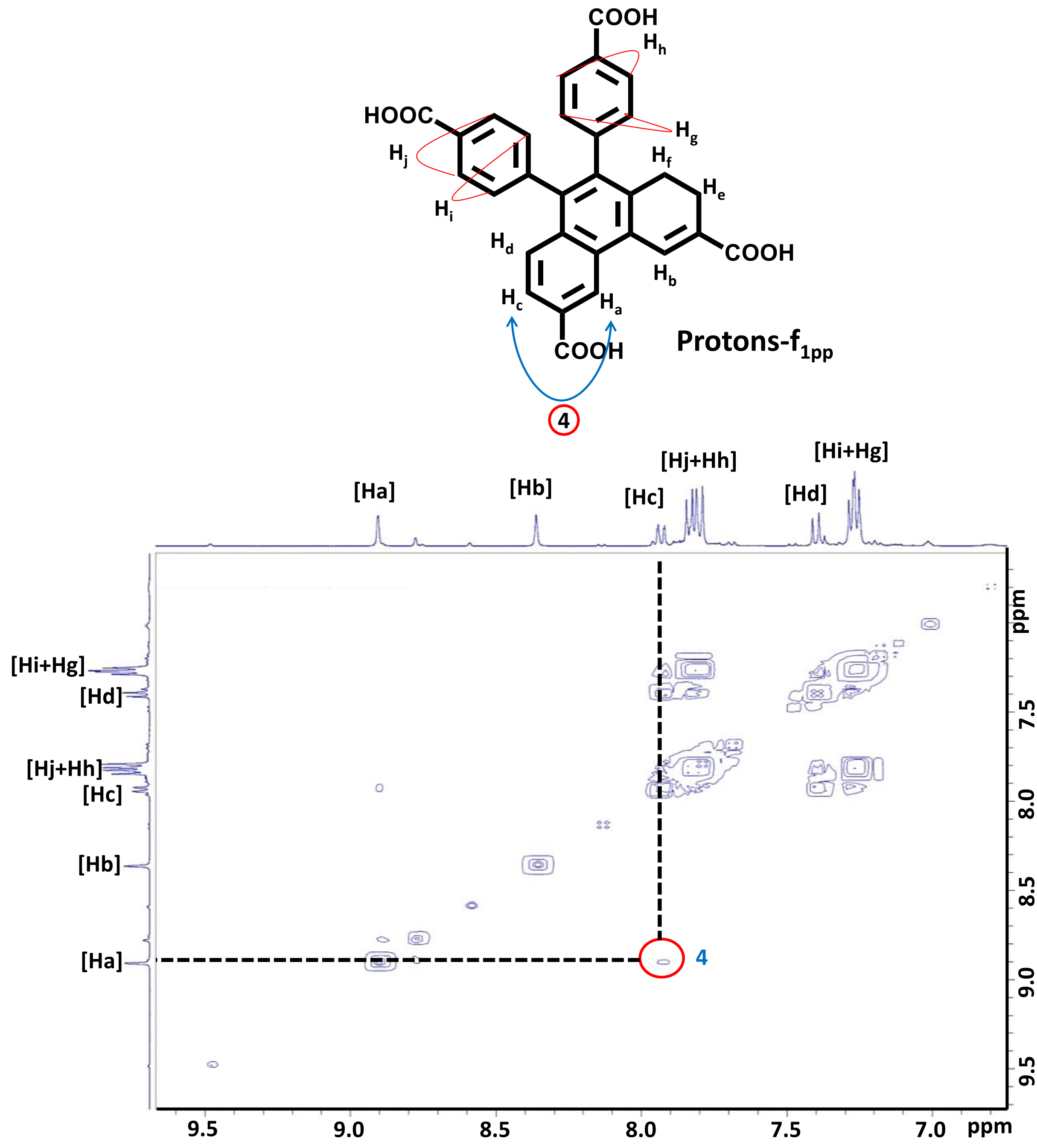

Figure S26. COSY 2D-NMR spectra showing correlation between ^{1}H-nuclei of $\mathbf{f_{1pp}}$ in DMSO-d_6 (contours have been circled in red with correlation in black dashed lines)

Explanation: Weak meta coupling between H_c and H_a. 4 bond coupling in downfield region also signifies aromaticity in one part of ring.

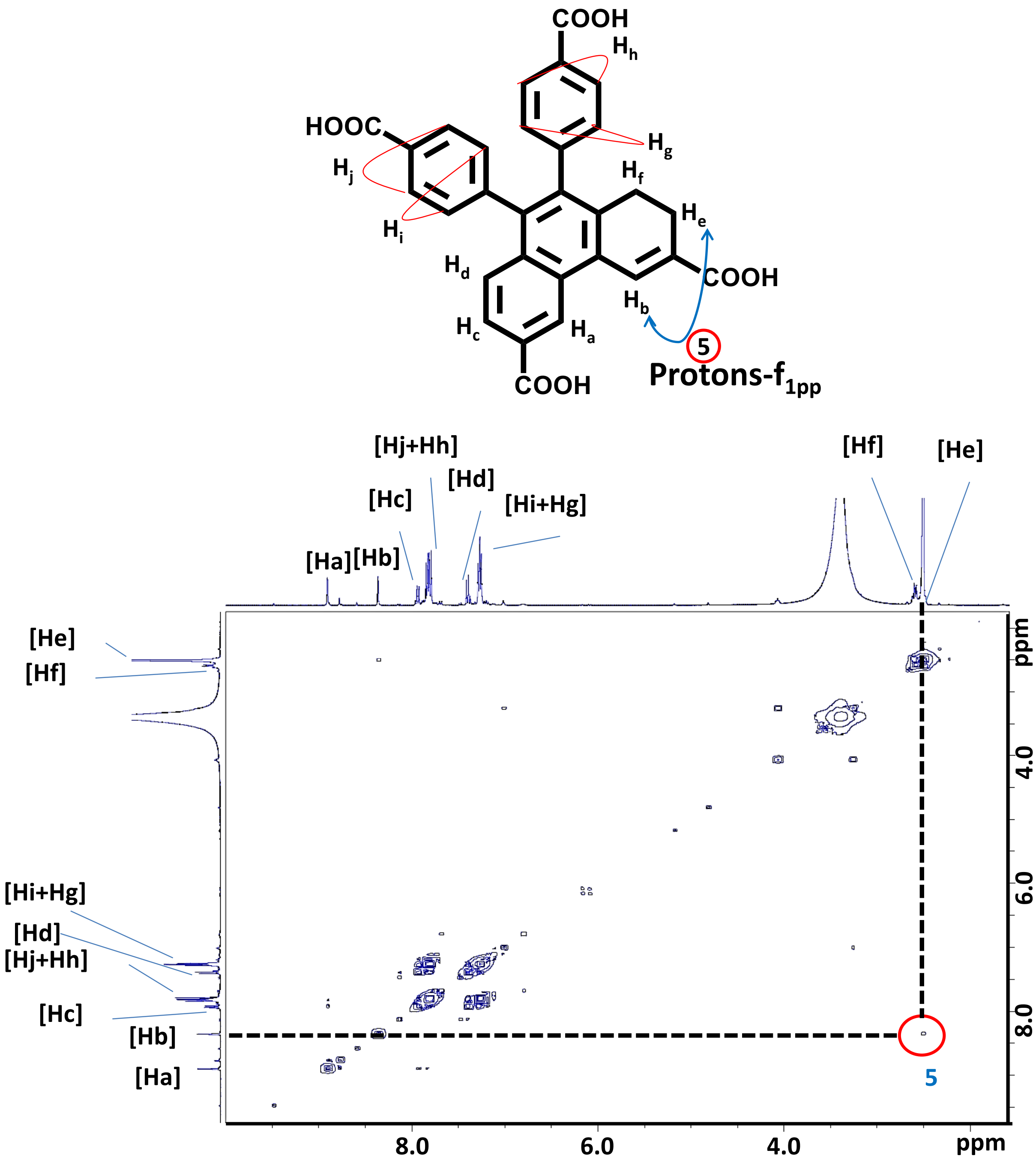

Figure S27. COSY 2D-NMR spectra showing correlation between ^{1}H-nuclei of $\mathbf{f_{1pp}}$ in DMSO-d_6 (contours have been circled in red with correlation in black dashed lines)

Explanation: Weak allylic coupling between H_e and H_b. 4 bond coupling in upfield region, also signifies lack of aromaticity in another part of ring. H_e and H_f coupling is not visible perhaps being very close to the diagonal peaks.

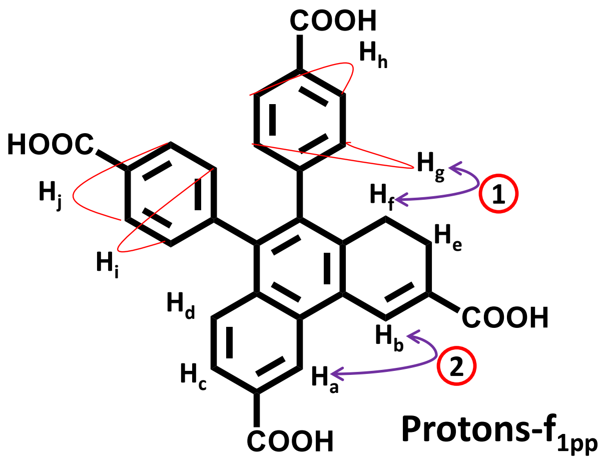

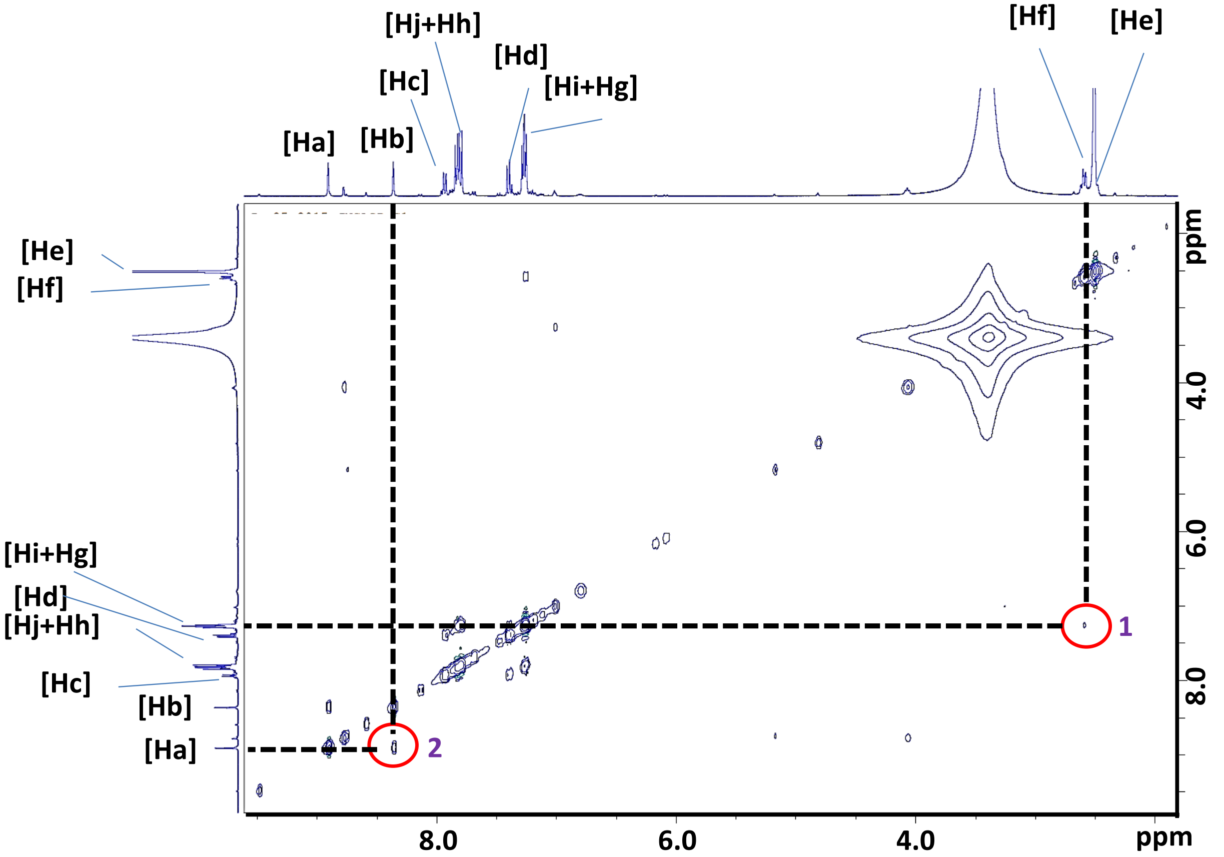

Figure S28. NOESY 2D-NMR spectra showing correlation between ^{1}H-nuclei of $\mathbf{f_{1pp}}$ in DMSO-d_6 (contours have been circled in red with correlation in black dashed lines)

Explanation: H_i-H_d coupling too close to diagonal peaks, hence perhaps not observed. Clear (H_a, H_b) and (H_f, H_g) NOE couplings confirm the proposed structure.

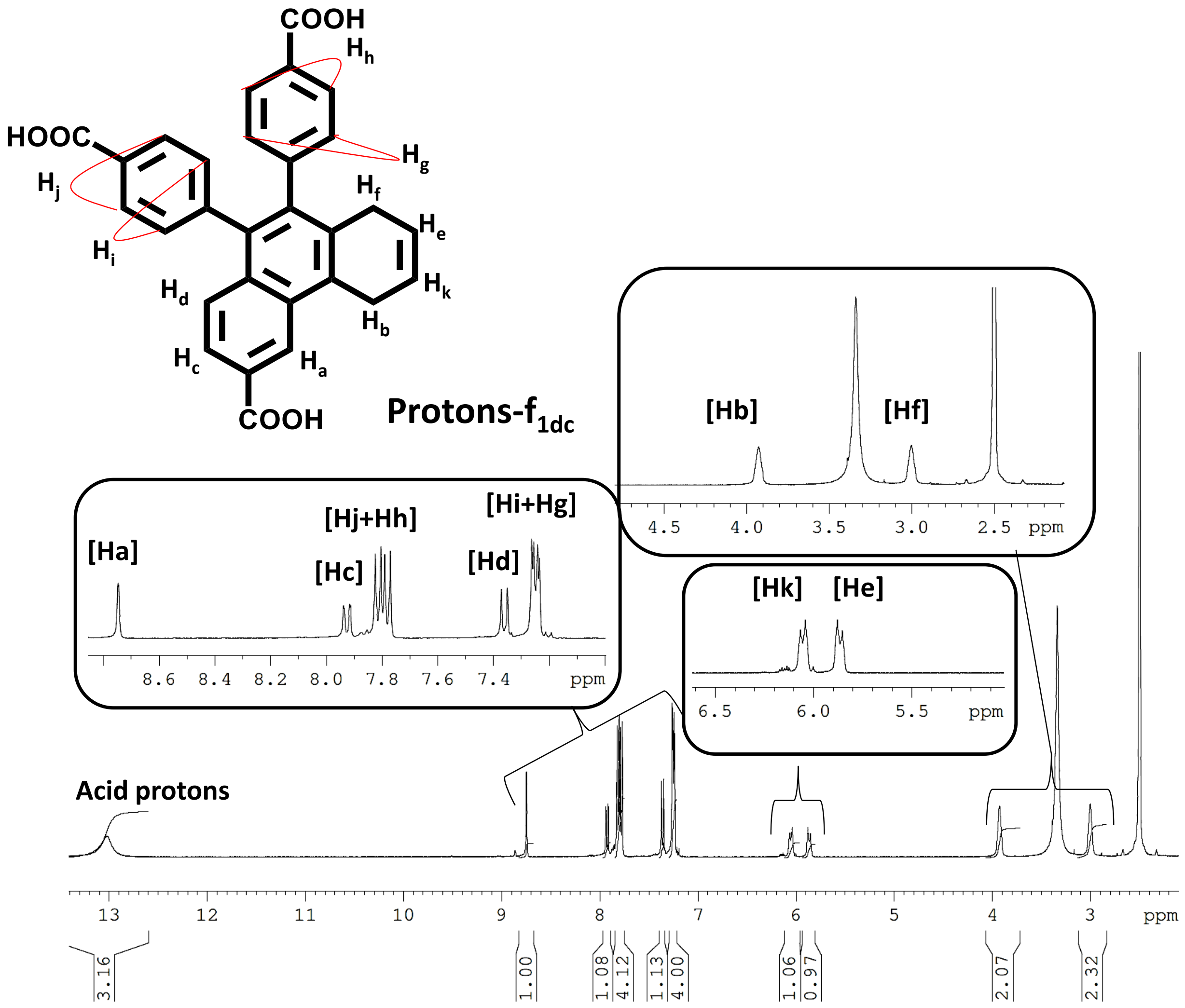

Figure S29. ^{1}H-NMR of $\mathbf{f_{1dc}}$ in DMSO-d_6

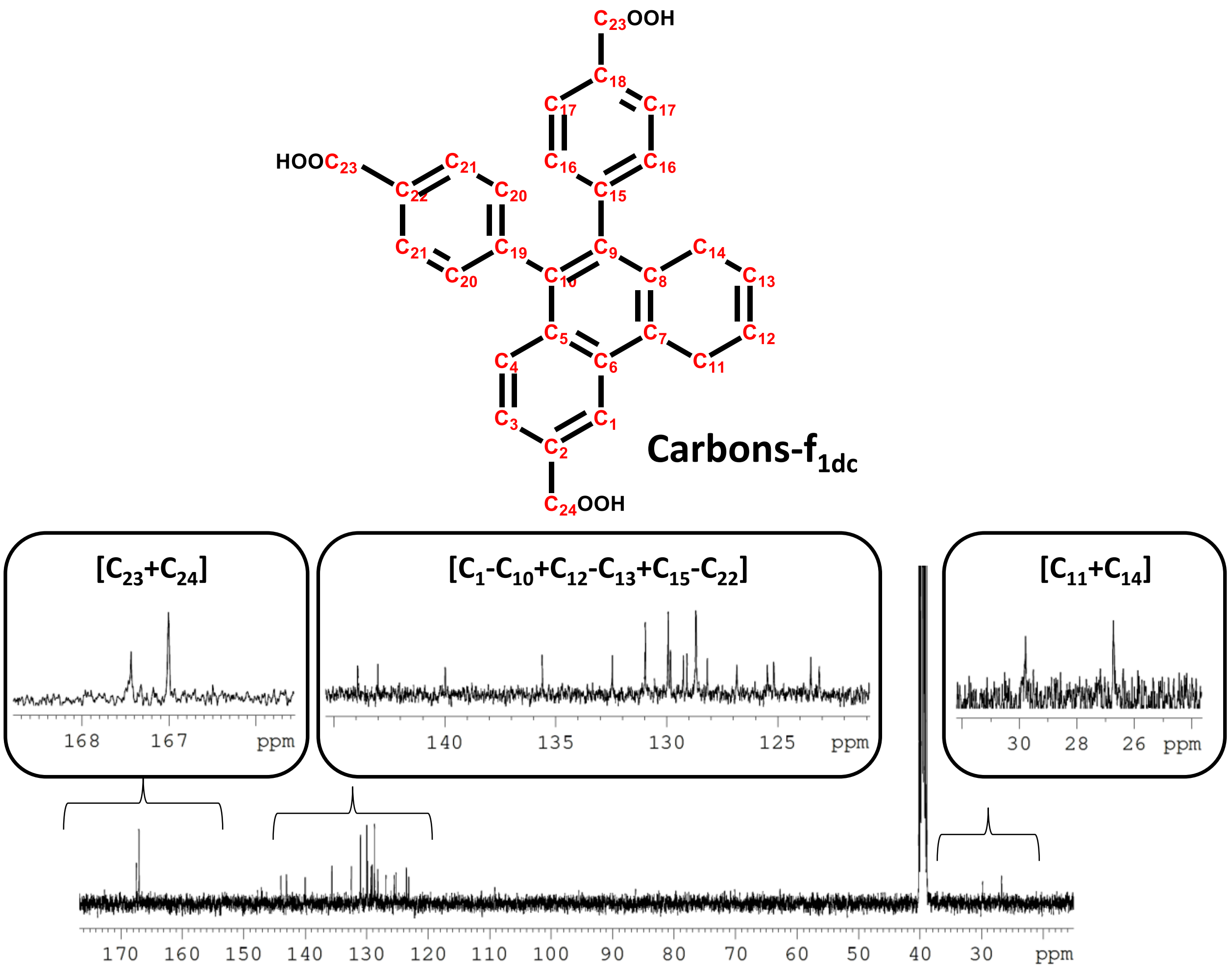

Figure S30. ^{13}C-NMR of $\mathbf{f_{1dc}}$ in DMSO-d_6 .

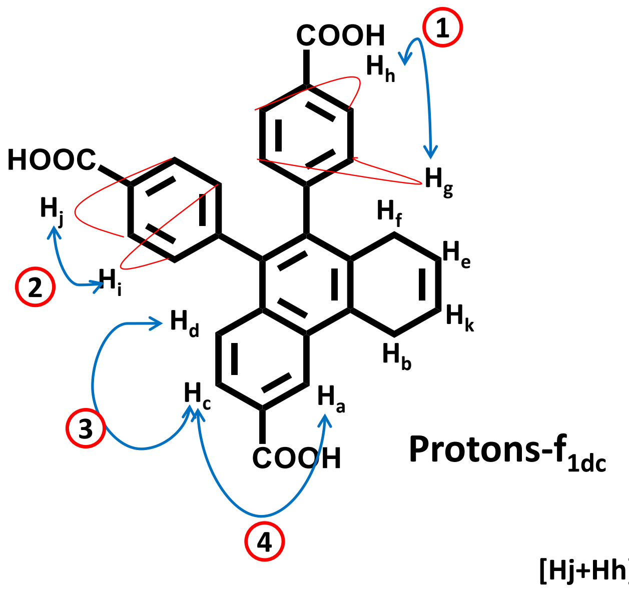

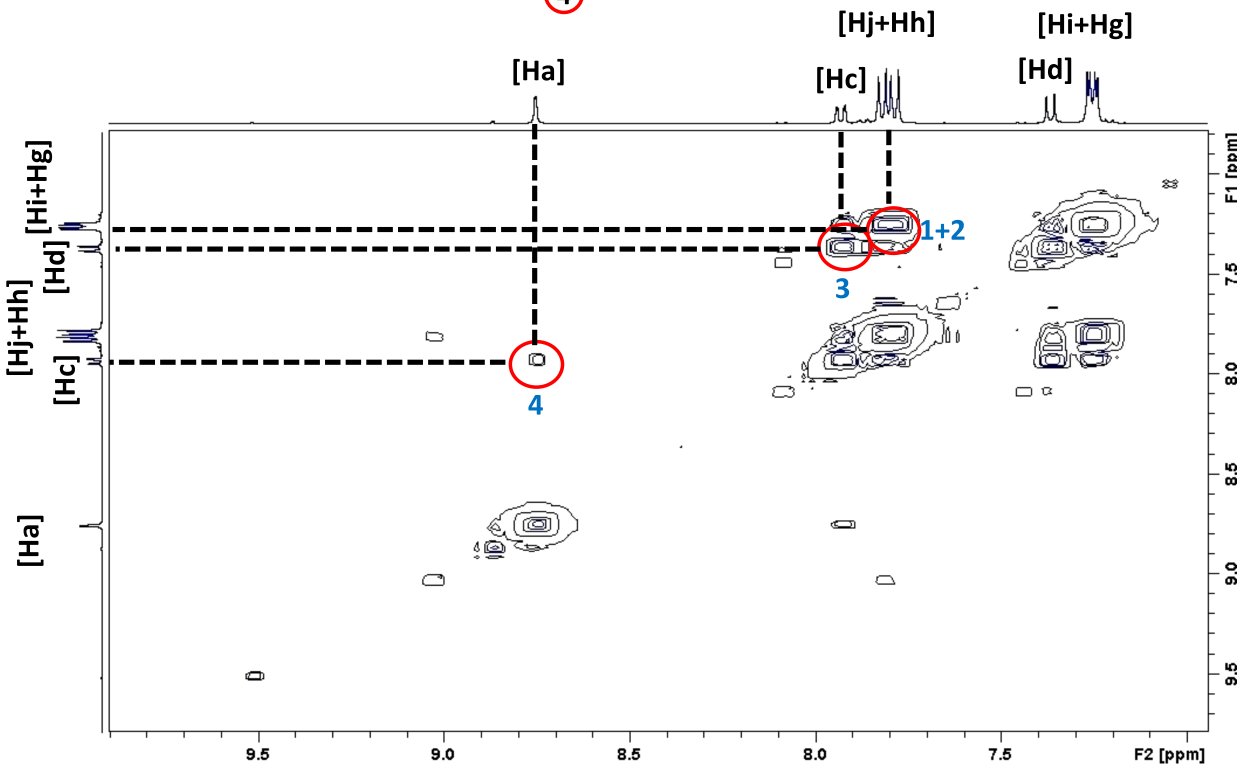

Figure S31. ^{1}H-COSY of $\mathbf{f_{1dc}}$ in DMSO-d$_6$ (contours have been circled in red with correlation in black dashed lines).

Explanation: 1,2 and 3 are three bond couplings in down field region. 4 is the weak four bond meta-coupling between H_a-H_c signifying aromaticity in one part of the molecule.

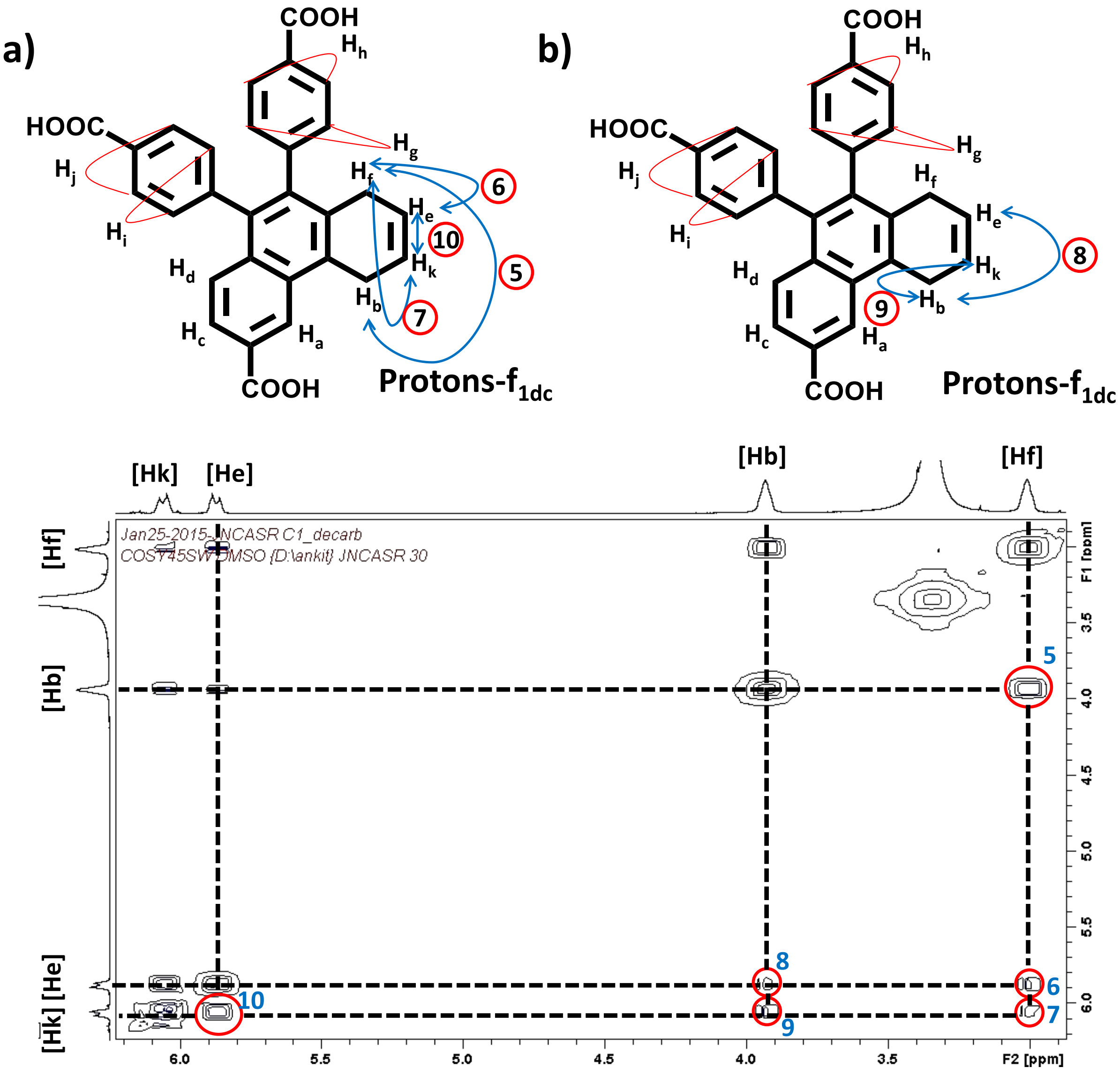

Figure S32. ^{1}H-COSY of $\mathbf{f_{1dc}}$ in DMSO-d$_6$ (contours have been circled in red with correlation in black dashed lines).

Explanation: Strong coupling between H_f and H_b (5) is a result of strong homo-allylic coupling which is characteristic of 1,4 cyclohexene systems. 6 and 9 are respective three bond couplings in sets (H_f, H_e) and (H_b, H_k). Similarly, 10 is a three bond coupling between H_k and H_e. 7 and 8 are allylic couplings in sets (H_f, H_k) and (H_b, H_e). downfield signifies lack of aromaticity in another part of ring.

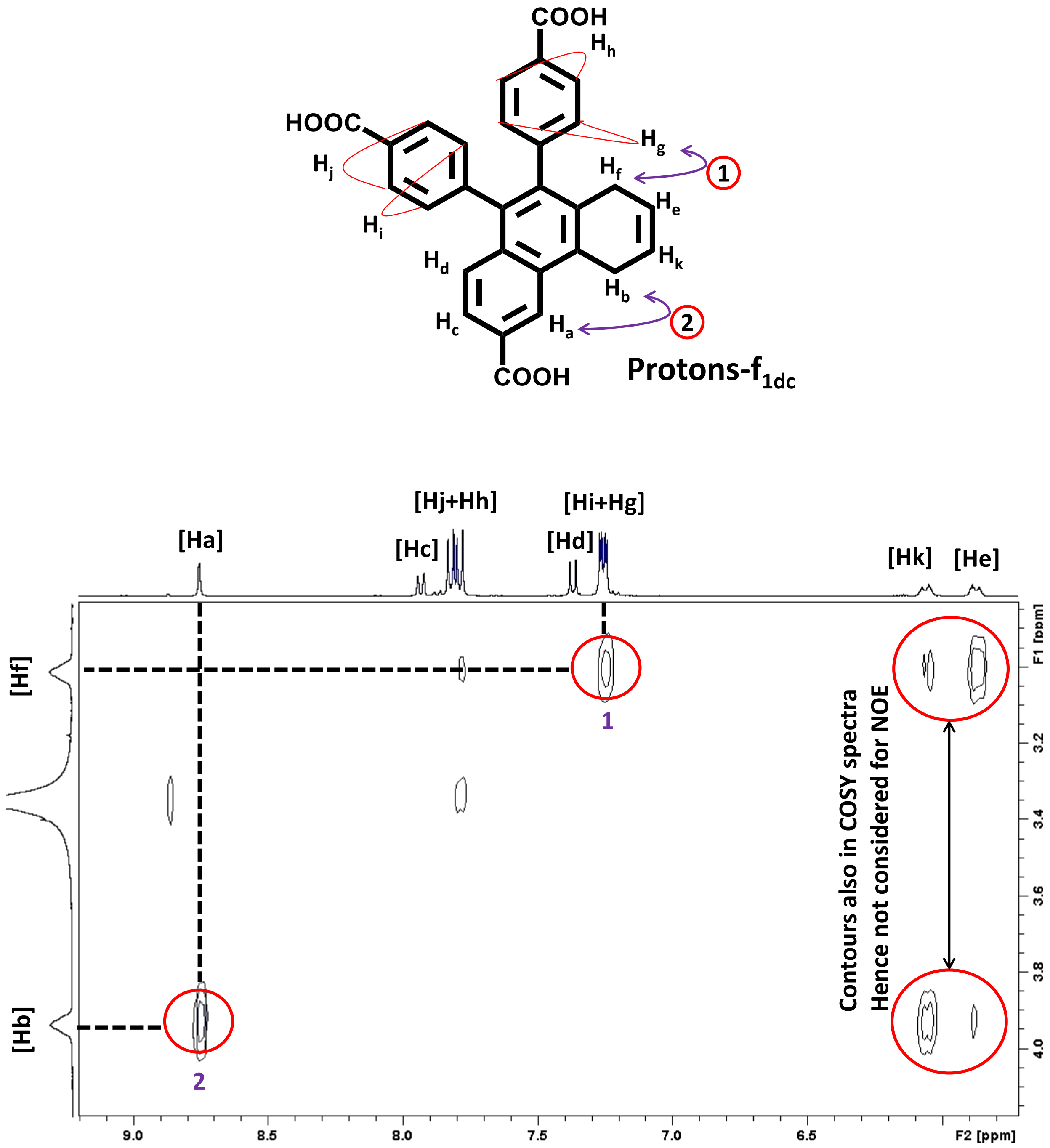

Figure S33. [1]H-NOE of $\mathbf{f_{1dc}}$ in DMSO-d_6 (contours have been circled in red with correlation in black dashed lines)

***Explanation*:** H_i-H_d coupling too close to diagonal peaks, hence perhaps not observed. Clear (H_a, H_b) and (H_f, H_g) NOE couplings confirm the proposed structure.

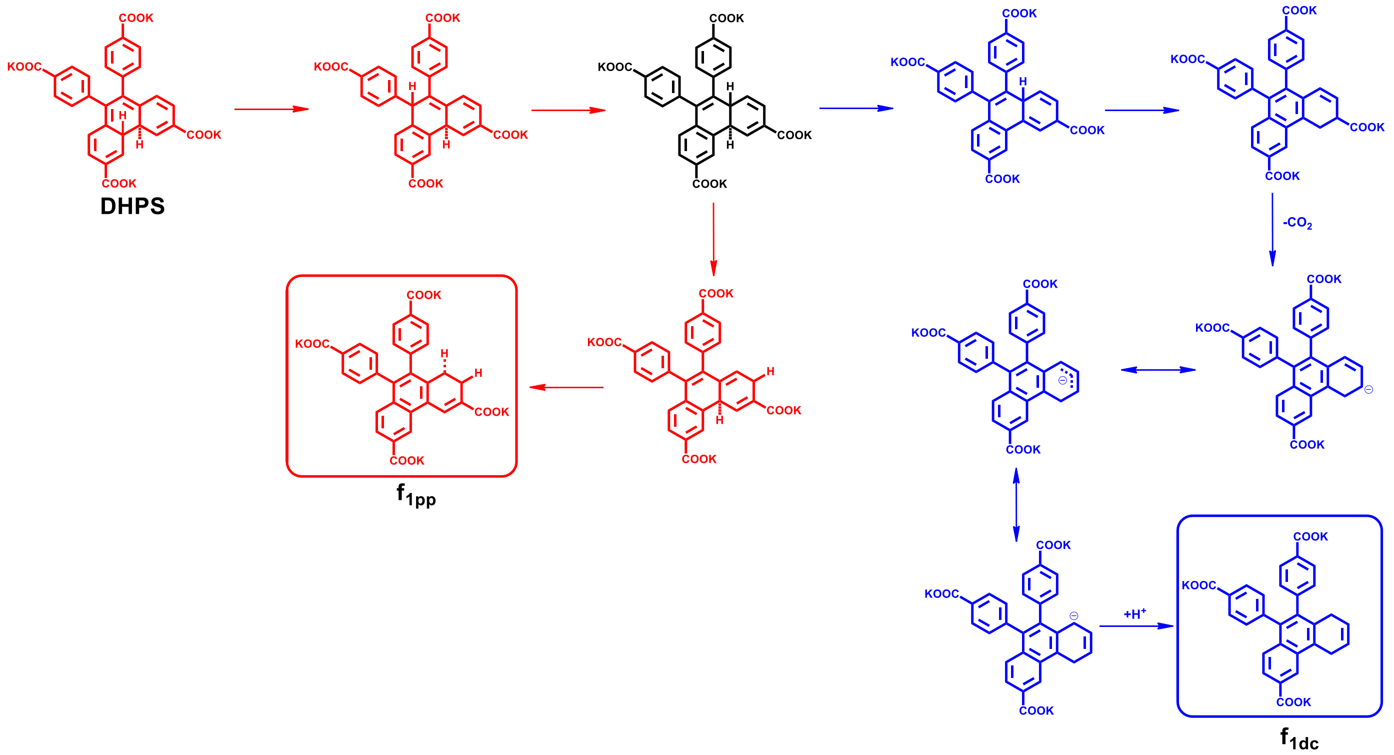

Figure S34. Proposed reaction pathway for $\mathbf{f_{1pp}}$ and $\mathbf{f_{1dc}}$ from **DHPS**. Hence **[DHPS] = [$\mathbf{f_{1pp}}$] + [$\mathbf{f_{1dc}}$].**

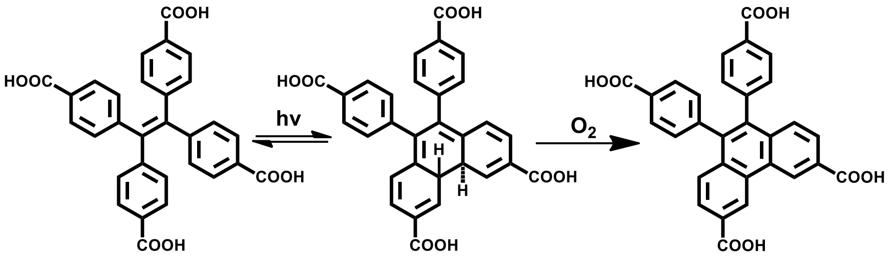

[M] [M] [M-2]

$$A \xrightarrow{k_1} B \xrightarrow{k_2} C$$

$$\frac{d[A]}{dt} = -k_1[A] \quad \text{(1)}$$

$$\frac{d[B]}{dt} = k_1[A] - k_2[B] \quad \text{(2)}$$

$$\frac{d[C]}{dt} = k_2[B] \quad \text{(3)}$$

$$[A] = [A]_o\, exp(-k_1 t) \quad \text{(4)}$$

$$[B] = \frac{k_1}{k_2 - k_1} \{exp(-k_1 t) - exp(-k_2 t)\}[A]_o \quad \text{(5)}$$

$$[C] = \left(1 + \frac{k_1 exp(-k_2 t) - k_2 exp(-k_1 t)}{k_2 - k_1}\right)[A]_o \quad \text{(6)}$$

Figure S35. Kinetic equations showing the expression for a series reaction, boxed reaction is the concentration profile of the intermediate.[a]

[a] Arnaut, L.; Formosinho, S.; Burrows, H. in *Chemical Kinetics From Molecular Structure to Chemical Reactivity*, *Ed. 1*, Elsevier Science, **2006**, pp. 86-87.

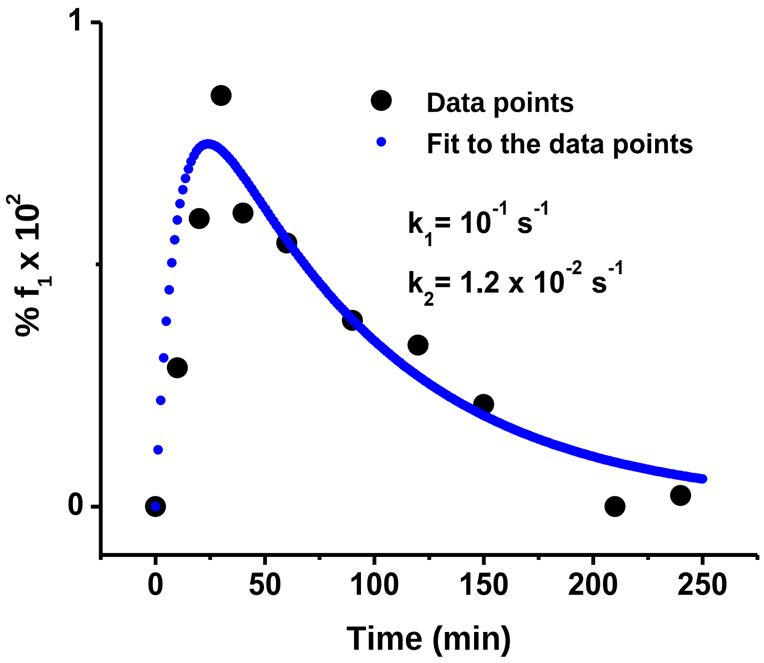

Figure S36. Intensity trace at 400 nm of the photo-oxidation profile of **TPTS** and **AC** (*10^{-4}M + 0.9 wt%, l = 1 cm, λ_{exc} = 350 nm, water*) under 254 nm irradiation with a graphical fit to equation 5 shown in figure S35.

***Explanation*:** Figure S36 shows the profile of intermediate $\mathbf{f_1}$ with varying time. This has been plotted from the emission trace followed at 400 nm of the **TPTS** and **AC** conjugate as it was being irradiated (Figure 2f). Trace at 400 nm in figure 2f actually follows the formation of $\mathbf{f_{1pp}}$ and $\mathbf{f_{1dc}}$ which are derivatives formed from **DHPS**. Calculating the net percentage of $\mathbf{f_{1pp}}$ and $\mathbf{f_{1dc}}$ formed from HPLC chromatograms at various stages of the irradiation the emission trace was normalized and hence fitted to equation 5 (Figure S35).

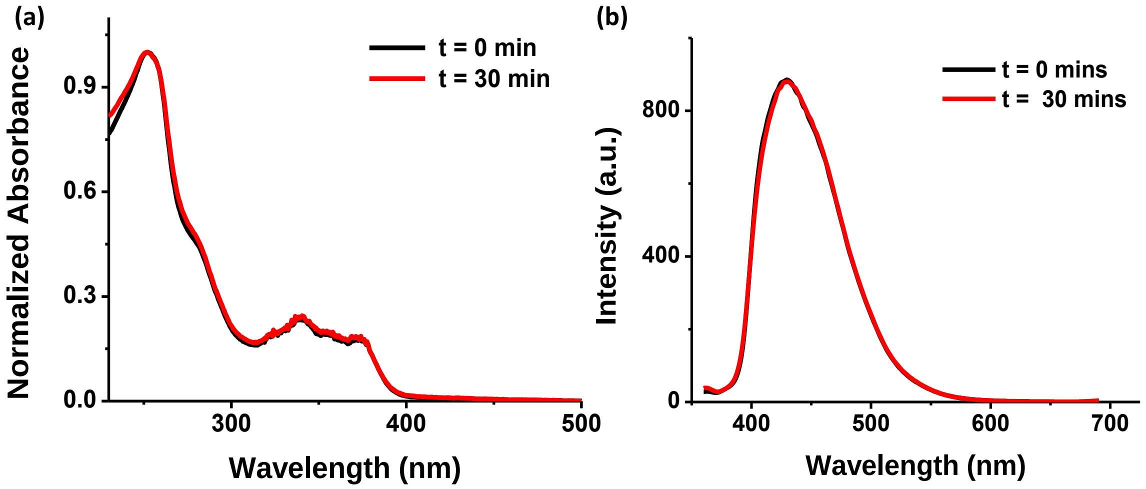

Figure S37. a) and b) UV absorption and emission spectra respectively of $\mathbf{f_{1pp}}$ under varying exposure times of oxygen gas.

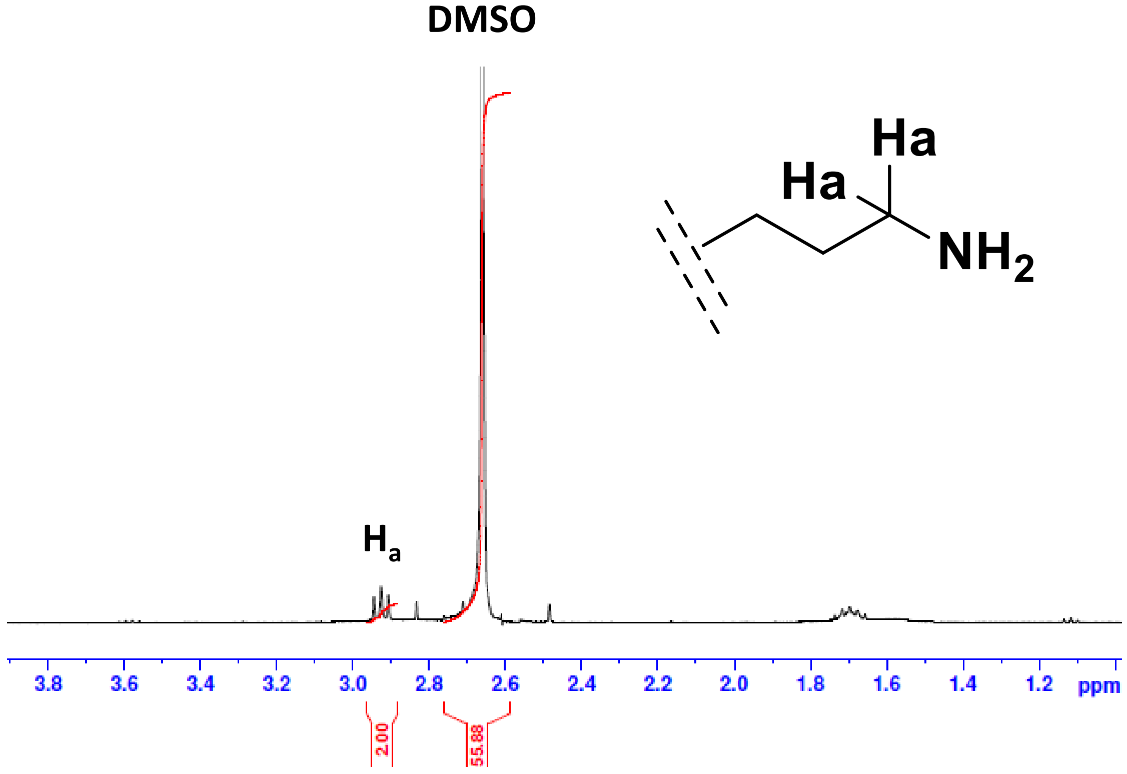

Figure S38. ^{1}H-NMR spectra of **AC** in D_2O with DMSO as an internal standard. Calculated amount of propyl amine chains is 0.0129 M per 1 ml of 1 wt% **AC** solution.

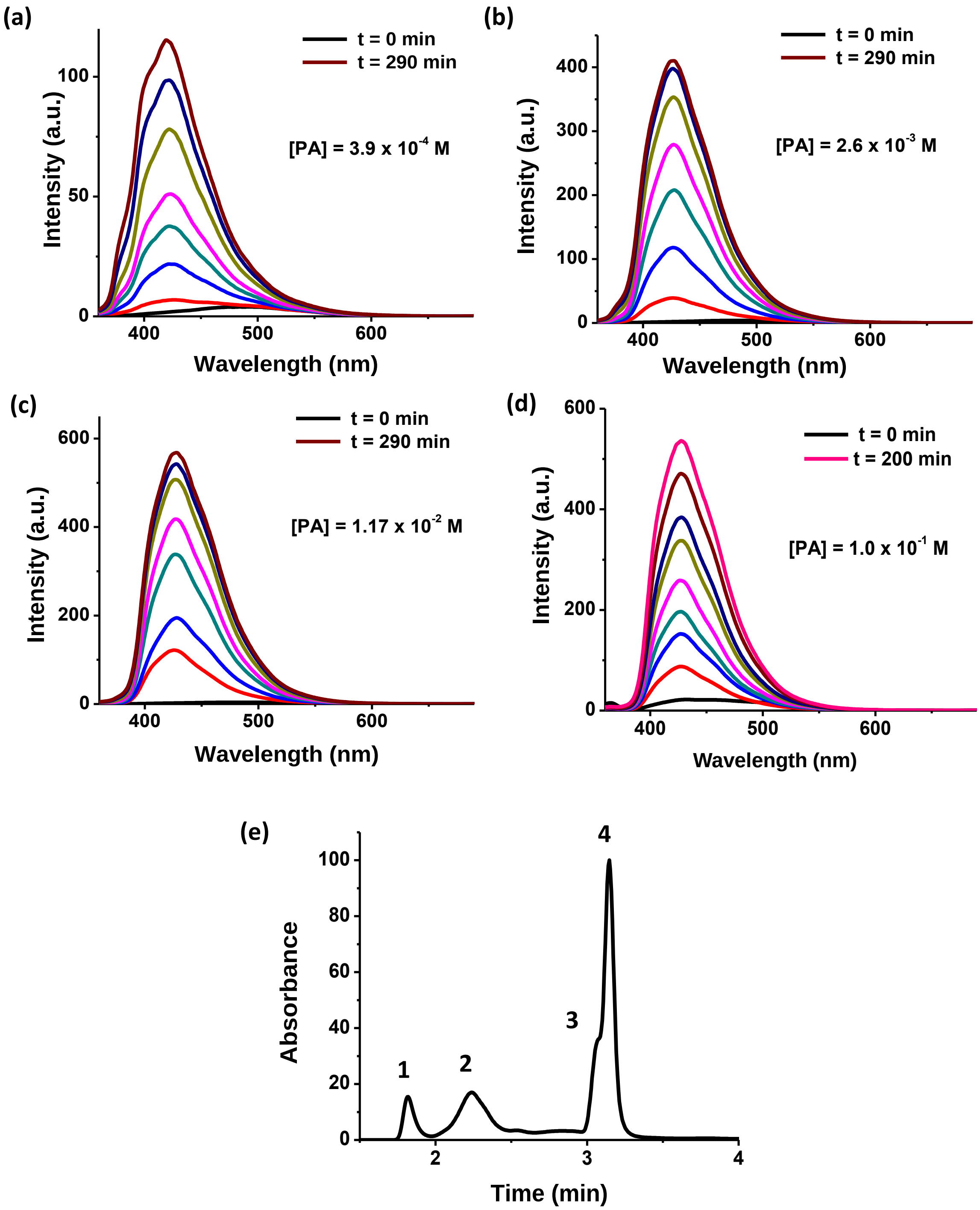

Figure S39. a) to d) Time dependent emission spectra showing photo-irradiation of **TPTS** in various concentrations of propyl amine [PA], inset showing the concentrations and e) HPLC trace of the saturated fraction after irradiation.

Explanation: The effect of propyl amine chains alone, on the photo-chemistry of stilbene derivatives, causing base induced tautomerization had been reported. Therefore we first tried to quantify the amount of propyl amine chains that are available to us in current concentration of AC. ^{1}H-NMR of **AC** in presence of a known concentration of DMSO as an internal standard showed that 1 wt% of amino clay contains around 1.2 X 10^{-2} M propyl amine chains (figure S38). We further carried out the irradiation of **TPTS** in presence of varying amounts of *n*-propyl amine which correspond to amino clay concentrations in which the transformation is seen (figure S39(a-c)). Also, to compensate for the multivalency argument in amino clay we also did the photochemistry in presence of 10 times higher concentration of propyl amine (0.1 M) (figure S39(d)). Emission spectra were recorded over time for these photo-irradiations. LC-MS measurement on the states where the reaction is saturated was also performed (figure 39(e)). Kinetics of these transformations was far too slow to be compared with AC (figure S41-42) to have any significant impact on the reaction outcome (*vide infra*). A plot of reaction rates observed over various orders of propyl amine concentration also do not show any trend to reach up to the value obtained in AC conjugate (figure S42) (*vide infra*).

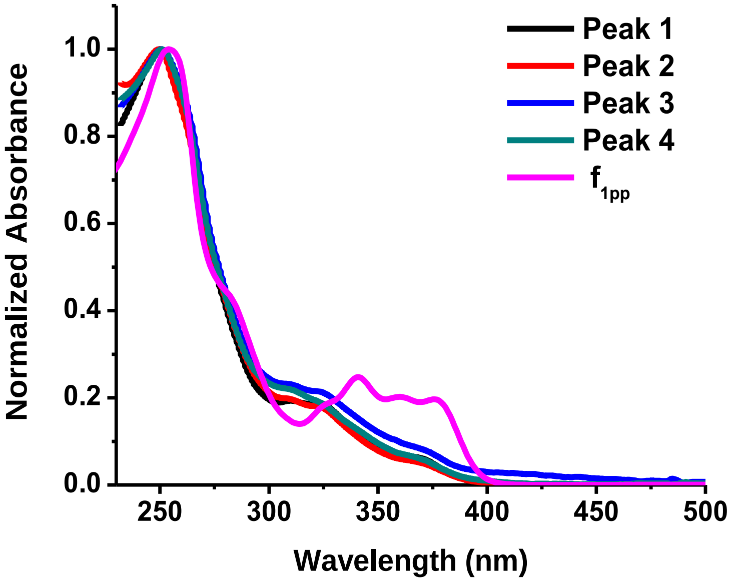

Figure S40. UV absorption spectra of various HPLC peaks in figure S39(e) as compared to the absorption of $\mathbf{f_{1pp}}$ (pink).

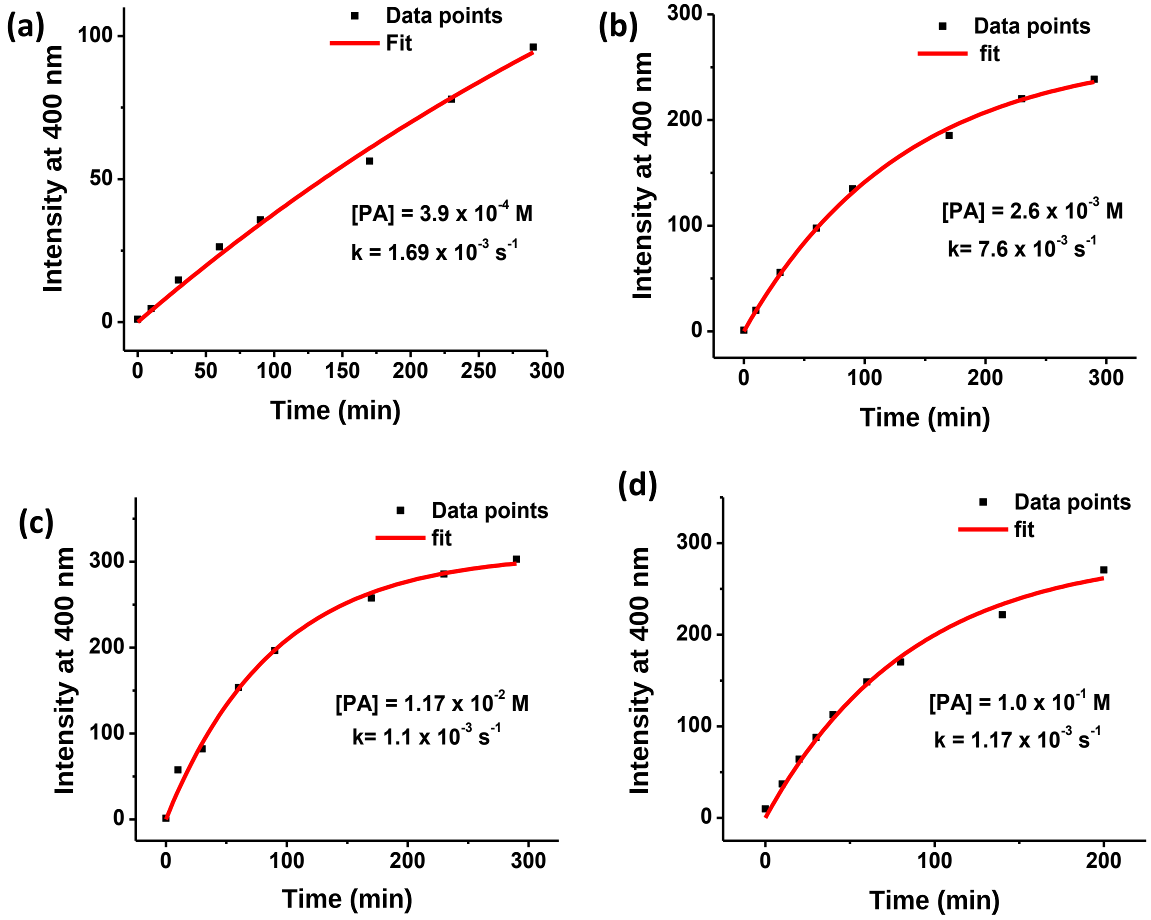

Figure S41. a) to d) Time dependent trace at 400 nm showing photo-irradiation of **TPTS** in various concentrations of propyl amine [PA], inset showing the concentrations and the calculated rate constants.

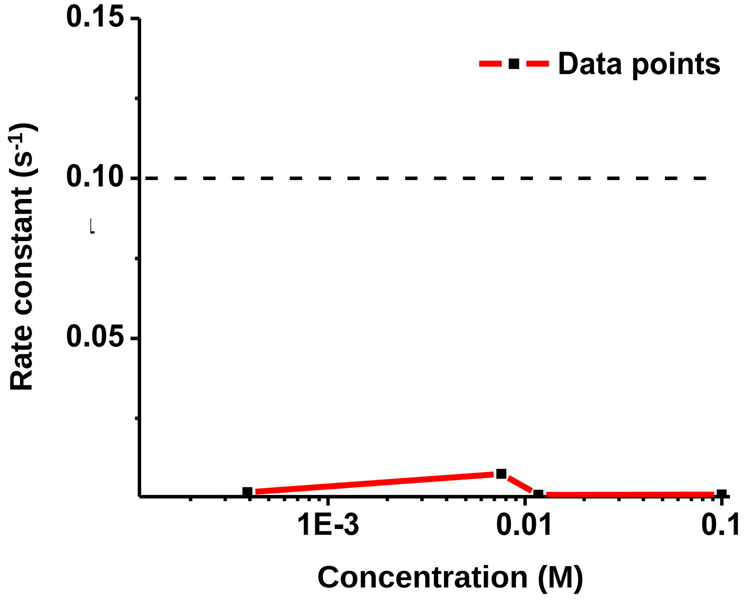

Figure S42. Plot of rate constants with varying propyl amine [PA] concentrations. The dotted line represents the value of k_1 in **TPTS** and **AC** solution
